# Follicular Helper T (T_FH_) Cell Targeting by TLR8 Signaling For Improving HBsAg-Specific B Cell Response In Chronic Hepatitis B Patients

**DOI:** 10.3389/fimmu.2021.735913

**Published:** 2021-08-26

**Authors:** Natarajan Ayithan, Lydia Tang, Susanna K. Tan, Diana Chen, Jeffrey J. Wallin, Simon P. Fletcher, Shyam Kottilil, Bhawna Poonia

**Affiliations:** ^1^Division of Clinical Care and Research, Institute of Human Virology, University of Maryland School of Medicine, Baltimore, MD, United States; ^2^Clinical Research, Gilead Sciences Inc., Foster City, CA, United States

**Keywords:** toll-like receptor 8, chronic hepatitis B, selgantolimod (SLGN), follicular helper T cell, B cell, HBsAg-specific B cell response, inflammatory cytokine, activation induced marker (AIM)

## Abstract

Identifying signaling pathways that induce B cell response can aid functional cure strategies for chronic hepatitis B infection (CHB). TLR8 activation with ssRNA was shown to enhance follicular helper T cell (T_FH_) function leading to improved B cell responses *in vitro*. We investigated whether this mechanism can rescue an exhausted immune response in CHB infection. Effect of TLR8 agonism on supporting cytokines and T_FH_ and B cells were evaluated using *ex vivo* and *in vitro* assays. The ability of an oral TLR8 agonist to promote T_FH_ and B cell response was tested in samples from phase 1b clinical trial. TLR8 agonism induced T_FH_ polarizing cytokine IL-12 in monocytes. Treatment of peripheral blood mononuclear cells (PBMCs) from CHB patients with TLR8 agonists induced cytokine IL-21 by T_FH_ cells with enhanced IL-21^+^BCL-6^+^ and ICOS^+^BCL-6^+^ co-expression. Mechanistically, incubation of isolated naïve CD4^+^ T cells with TLR8 triggered monocytes resulted in their differentiation into IL-21^+^ICOS^+^BCL-6^+^ T_FH_ in an IL-12 dependent manner. Furthermore, co-culture of these IL-21 producing T_FH_ with autologous naïve B cells led to enhanced memory (CD19^+^CD27^+^) and plasma B cell generation (CD19^+^CD27^++^CD38^+^) and IgG production. Importantly, in T_FH_ from CHB patients treated with an oral TLR8 agonist, HBsAg-specific BCL-6, ICOS, IL-21 and CD40L expression and rescue of defective activation induced marker (AIM) response along with partial restoration of HBsAg-specific B cell ELISPOT response was evident. TLR8 agonism can thus enhance HBV-specific B cell responses in CHB patients by improving monocyte-mediated T_FH_ function and may play a role in achieving HBV functional cure.

**Graphical Abstract:**
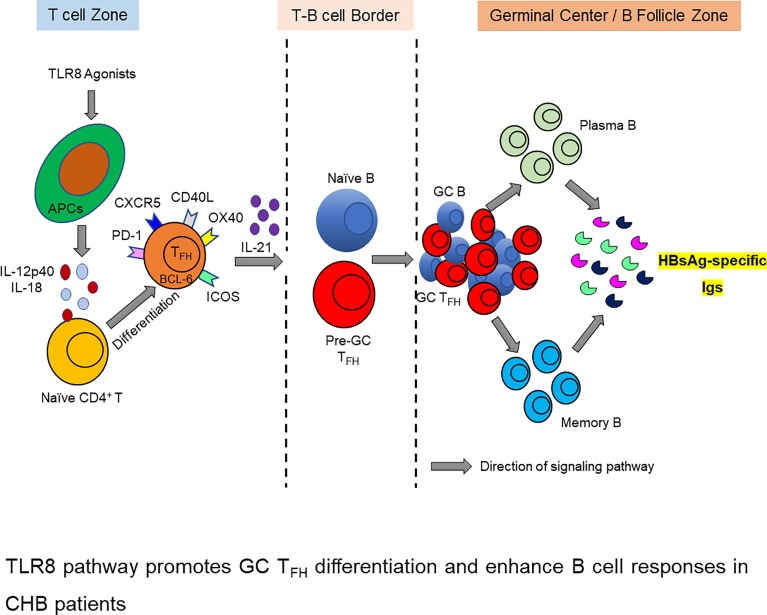


## Introduction

Hepatitis B virus (HBV) infection remains a major global health burden with over 257 million people worldwide chronically infected. A major barrier to HBV cure with standard nucleos/tide analog (NUC) therapy is the persistent dysfunction of the antiviral (HBV) immune response despite therapy induced viral suppression (1, 2). As such identifying immunomodulatory strategies that aid HBs antigen loss/seroconversion, which defines functional cure of infection, is of high significance in HBV cure research (3).

In the past years, HBs antigen (HBsAg)-specific B cells have received attention and several laboratories including ours identified dysfunction in antigen-specific B cell response (4–6). These B cells have upregulated inhibitory receptor expression and impaired HBsAg-specific IgG production *ex vivo*. Dysfunctional B cell response can be attributed to intrinsic B cell defects along with insufficient help from CD4^+^ T cells. T follicular helper (T_FH_) cells are the CD4^+^ T cells that provide signals for antigen specific B cell maturation into a plasma cell, making them essential for the generation of most isotype switched and affinity matured antibodies. Coordinated T_FH_ and B cell response determines acquisition and maintenance of antibody response to prophylactic HBsAg based vaccine (7). This response is defective during CHB infection, where an accumulation of activated and phenotypically abnormal T_FH_ dysregulates cytokine profiles (8, 9). Dysfunctional antigen-specific T_FH_ response was shown to promote persistence of HBV in a mouse model (9). In samples from CHB patients, impaired T_FH_ response was shown to be due to IL-21 suppression mediated by CTLA-expressing Tregs, which could be restored by inhibiting Tregs with an antibody against CTLA-4 (9). We showed that abnormal T_FH_ persist even in long-term NUC treated CHB patients, possibly explaining the low incidence of anti-HBs seroconversion in these patients (6). Thus, we believe this impaired T_FH_ response is a critical defect associated with hepatitis B virus persistence and resolving the dysfunction will be of significance in resolution of CHB.

Among the immunomodulatory strategies, toll-like receptors are a promising way to correct immune deficiencies. Human liver derived monocytes respond strongly to TLR8 agonism, producing IL-12 and IL-18 (10). IL-12 induced by TLR8 signaling has a potential impact on anti-viral CD8 T cell response (11). Importantly, IL-12 was shown to aid T_FH_ differentiation that led to plasma cell generation in individuals vaccinated with a live attenuated vaccine (12). We investigated whether similar mechanism can restore dysfunctional T_FH_ response and aid exhausted HBsAg-specific B cell response (4–6) present in chronic HBV infection. Here we demonstrate that TLR8 signaling induces differentiation of T_FH_ cells into efficient B cell helpers with potential to promote plasma cell generation and an HBsAg-specific B cell response.

## Materials and Methods

### Patients and Samples

For investigating *in vitro* effects of TLR8 stimulation, peripheral blood samples were available from CHB patients or HBV vaccinated healthy volunteers enrolled in HOPE cohort at University of Maryland, Baltimore. The study protocol was approved by the institutional ethical committee, and all subjects gave written, informed consent. Demographic and clinical details of patients are provided in **Table 1**.

**Table 1:** Demographic and clinical characteristics of chronic hepatitis B patients.

Clinical Characteristics	Chronic Hepatitis B (CHB) Patients
Subjects	n=29
HBV DNA (copies/mL)	UND (11), <20 (9), >7906 (9)
Hgb (gm/dL)	14.4
Platelet (10^9^/L)	212
Creatinine (mg/dL)	0.8
ALT (U/L)	30
AST (U/L)	26
Albumin (g/dL)	4.6 (14), ND (15)
HBsAg IU	UND (2), 11974 (18), ND (9)
HBeAg	positive (8), negative (20)
HBsAg	positive (27), negative (2)
HBeAb	positive (20), negative (8), ND (1)
Anti-HBs	negative (27), ND (2)

Values are presented as median. ALT, Alanine aminotransferase; AST, Aspartate aminotransferase; UND, Undetected; ND, Not Detected.

For investigating *in vivo* effects of TLR8 agonism, peripheral blood samples from previously completed phase 1b clinical trial (ACTRN identifier: 12617000235303) of a selective-TLR8 agonist (Selgantolimod (SLGN), GS-9688) in CHB patients (N=14) and in healthy subjects (13, 14) were available courtesy of Gilead Sciences. Paired samples from baseline (BL) and TLR8 single oral dose treatment (8 hours-post SLGN, 1.5 or 3 mg) time-points were used here.

### TLR Agonists Stimulation of PBMCs

The following TLR ligands (InvivoGen, each 1 µg/mL) were added into wells of 24-well plate containing 1-2 x 10^6^ PBMCs: Poly(I:C) HMW (TLR3), LPS-EK Ultrapure (lipopolysaccharide, TLR4), Imiquimod (IMQ) (R837, TLR7), Resiquimod (R848, TLR7/8), TL8-506 (TLR8), ssRNA40/LyoVec (TLR8) and CpG-ODN (TLR9). The concentration of 1 µg/mL is within the range of manufacturer recommendation and induced maximum cytokine response (not shown) (10, 12). Golgi plug (1 µl/mL, BD Biosciences) was added to block the cytokines release and cell culture continued for 18h. For some experiments, PBMCs were stimulated with agonists alone or in combination with recombinant human HBsAg subtype adw (10 µg/mL, Fitzgerld) (15) and PepMix HBV (LEP) Ultra (2 µg/mL, JPT) and cultured for 5 days at 37°C incubator. In experiments for transcription factor induction, cells were re-stimulated with Phorbol-12-myristat-13-acetate (PMA, 50ng/mL) and Ionomycin (Ion, 1 µg/mL) on day 4.

### Flow Cytometry (FACS) and Intracellular Cytokine Staining (ICS)

PBMCs were stained and analyzed by flow cytometry using standard methods (panels listed in **Supplementary Table 1**). Cells were acquired on a Cytek Aurora multi-color FACS machine and data analysis done by Flow Jo software (Tree Star, San Carlos, California, USA).

### Activation Induced Markers (AIM) Assay

For examination of contribution of TLR8 signaling in HBV antigen-specific germinal center like (GC) T_FH_ induction, a cytokine-independent approach was followed (16, 17). It is known that formulation of antigen can influence the type and extent of activation markers induced in CD4^+^ T cells (18). To capture multiple activation markers induced in T_FH_ with TLR8 signaling, here we used combination of HBsAg intact protein and peptide, as is described for other antigens (16, 17). Approximately 1-2 x 10^6^ PBMCs were aliquoted into 24-well plate and stimulated with HBV envelope peptide (2 µg/mL, JPT) and recombinant human HBsAg subtype adw (10 µg/mL, Fitzgerald). Intact protein antigens or antigenic peptides are used for stimulating antigen specific CD4 T cell response. Staphylococcal enterotoxin B (SEB 1 µg/mL, Toxins Technology) served as positive control and AIM-V medium alone served as untreated negative control (UT). CXCR5-BV605 and CXCR3-PeCy7 antibodies (Biolegend, 1 μl each) were added directly into the cell culture well and also included in the staining panel. After 18h stimulation, PBMCs cultures were washed twice with 1X PBS and followed the FACS staining protocol as stated in the above method using AIM assay panel flow antibodies provided in **Supplementary Table 1**. Cells were washed, fixed with 1% paraformaldehyde and acquired on the same day.

### Follicular Helper T Cells (T_FH_) Differentiation

To examine TLR8-mediated differentiation of T_FH_ cells, CD14^+^ monocytes and naïve CD4^+^ T cells were isolated from autologous human PBMCs of CHB patients (n=4) by negative selection (Miltenyi Biotec). The following co-culture experiments were adopted from previously published methods (12). First, purified CD14^+^ monocytes (1 x 10^6^ cells/mL) were left untreated or treated with TLR8-specific agonists ssRNA40/Lyovec (1 µg/mL, InvivoGen) or TL8-506 (1 µg/mL, InvivoGen) for 1.5 h. Isolated autologous naïve CD4^+^ T cells were directly added into the culture at a ratio of 2:1 (monocyte:CD4^+^) along with SEB (1 µg/mL, Toxins Technology). After 6 days, cells were either processed for T_FH_ cell sorting for further downstream study or re-stimulated overnight (O/N) with PMA (phorbol-12-myristat-13-acetate, 50 ng/mL, Sigma) and ionomycin (Ion 1 µg/mL, Sigma) as positive control to effectively activate T_FH_ cells. After 3-4h, Golgi plug (1 µl/mL, Invitrogen) was added and culture continued for additional 18h to investigate T_FH_ cell proliferation and differentiation related markers by flow cytometry. The culture supernatants were recovered and tested for IL-12p40, IL-18 and IL-21 cytokine quantifications by Luminex multiplex immunoassay.

### T_FH_ Cells Sorting and Naïve B Cells Co-Culture

For T_FH_ sorting, cells recovered after 6 days of monocyte-naïve CD4 co-culture (n=4) were stained with anti-human PD-1-BV421, ICOS-BV510, CXCR5-BV605, CXCR3-PeCy7 and CD4-APC antibodies. T_FH_ cell sorting was carried out on BD FACS Aria III.

For naïve B-T_FH_ co-culture, naïve B cells were isolated (Miltenyi Biotec) from autologous PBMCs of CHB patients (n=4) and co-cultured with sorted T_FH_ cells (from above) at 2:1 (B:T_FH_) in the presence of SEB (1 µg/mL) for 7 days after which T_FH_-mediated differentiation of B cells into various subsets was analyzed by flow cytometry.

### Neutralization (Ab Blocking) and Recombinant Protein Addition

Purified CD14^+^ monocytes (1 x 10^6^ cells/mL) (N=4) were pre-treated with LEAF purified mouse IgG1, k isotype control, Ultra-LEAF purified anti-human IL-12p40 or Ultra-LEAF purified anti-human IL-18 neutralizing antibodies (each 10 µg/mL, Biolegend) for 2h at 37°C humidified incubator (19), followed by stimulation with TL8-506 or ssRNA40/Lyovec for 1.5h. For recombinant protein stimulation assay, isolated autologous naïve CD4^+^ T cells were treated with recombinant human IL-12 or recombinant human IL-18 (each 20 ng/mL, Biolegend) followed by co-culture experiments as described above.

### B Cell ELISPOT Assay

PBMC from CHB patients obtained at baseline (BL, pre-TLR8) and TLR8 (8h post-Selgantolimod) single oral dose were tested. To examine HBsAg-specific antibody secreting B cells, cells were stimulated with polyclonal stimuli R848 (1 µg/mL, Mabtech) and recombinant human IL-2 (10 ng/mL, Mabtech) and cultured for 5 days to effectively induce memory B cell proliferation. B cell ELISPOT assay was conducted as previously described (6).

### Luminex Multiplex Immunoassay

Quantification of IL-12p40, IL-18 and IL-21 in cell culture supernatants collected from co-culture experiments was analyzed by Luminex multiplex immunoassay (Luminex LX200 multianalyte system, Bio-Rad Corporation) according to manufacturer protocol.

### Enzyme-Linked Immunosorbent Assay (ELISA)

Quantification of human class-switched immunoglobulin (IgG) was made in cell culture supernatants collected from naïve B-T_FH_ cell co-cultures using high sensitivity ELISA kit according to manufacturer’s protocol (XpressBio, Express Biotech International).

### Statistical Analyses

Statistical analyses were performed using one-way analysis of variance (ANOVA) or Kruskal-Wallis with Dunn’s multiple comparisons test. Paired samples between BL (pre) and TLR8 (8h-post) were analyzed by two-tailed paired, or the Wilcoxon signed-rank Student’s t test or non-parametric Mann-Whitney U test for comparisons between treatments. A p value of <0.05 was considered significant. Levels of significance are indicated by *: *p ≤ 0.05, **p ≤ 0.01, ***p ≤ 0.001, ****p ≤ 0.0001 and ns (no significance).

## Results

### *In Vitro* TLR8 Stimulation Induces Proinflammatory Cytokines in Monocytes From CHB Patients

**Figure 1:**
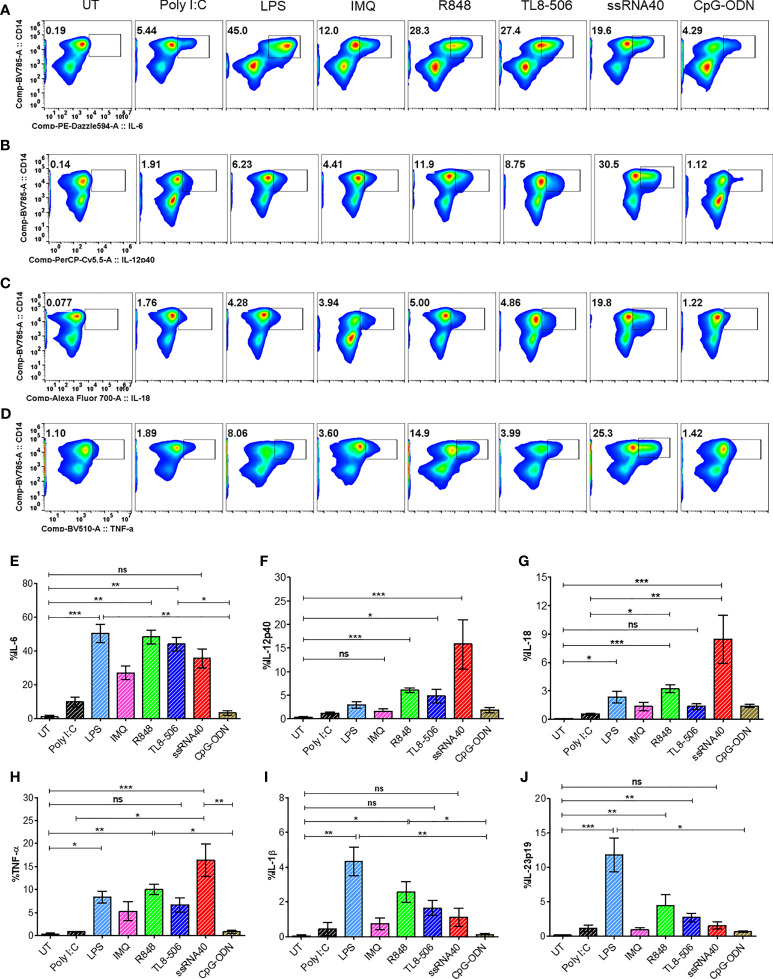
*In vitro* TLR8 stimulation augments proinflammatory cytokines in monocytes. PBMCs from CHB patients (n=6) stimulated with various TLR agonists as indicated to evaluate T_FH_ polarizing pro-inflammatory cytokine induction in monocytes. Representative pseudo color flow plots show intracellular expression of T_FH_ skewing pro-inflammatory cytokines **(A)** IL-6, **(B)** IL-12p40, **(C)** IL-18 and **(D)** TNFα, gated on CD14^+^HLA-DR^+^ monocytes. Numbers inside the box indicate percentages of CD14^+^HLA-DR^+^ monocytes secreting pro-inflammatory cytokine events compared between untreated (UT) negative control and different TLR agonists treated PBMCs. Frequencies shown in **(E)** IL-6, **(F)** IL-12p40, **(G)** IL-18, **(H)** TNFα, **(I)** IL-1β and **(J)** IL-23p19 as percentage of cytokine positive CD14^+^HLA-DR^+^ cells. Error bar on the graph represents the standard error of mean calculated from 6 individual donors. Differences between stimulations calculated by one-way analysis of variance (ANOVA) or Kruskal-Wallis test for multiple comparisons. P values ≤0.05*, 0.01**, 0.001*** indicate statistical significance levels. ns, no significance; IMQ, imiquimod; LPS, lipopolysaccharide.

TLR8 signaling induces IL-12 in monocytes, which supports T_FH_ differentiation in healthy individuals (12). We tested whether this pathway induces T_FH_ polarizing cytokines in samples from CHB patients. PBMCs isolated from CHB patients were stimulated with various TLR agonists for 18-24h or with medium alone (UT) as negative control. The ligation of TLR8 in monocytes by synthetic agonists, single stranded RNA40 (ssRNA40/LyoVec), TL8-506 and TLR7/8 ligand R848 induced proinflammatory cytokines IL-6, IL-12p40, IL-18, TNF-α in CD14^+^HLA-DR^+^ activated monocytes (**Figures 1A–D**) as analyzed by intracellular cytokine staining using flow cytometry. TLR8 agonist ssRNA40 significantly enhanced the production of cytokines IL-12p40 and IL-18 when compared with other TLR agonists. LPS stimulation induced cytokines IL-6, IL-1β and IL-23p19. Relative frequencies of CD14^+^HLA-DR^+^ monocytes producing these proinflammatory cytokines in response to various TLRs engagement are shown (**Figures 1E–J**). The frequencies of CD14^+^HLA-DR^+^ monocytes or the expression levels of the TLR8 receptor in CD14^+^HLA-DR^+^ monocytes did not change with any of the TLR agonist treatments (**Supplementary Figures 1A–C**). TGF-β1 was induced only by TL8-506 and no significant induction of IL-27p28 was observed (**Supplementary Figures 1D, E**). These data demonstrate that cytokines IL-12 and IL-18 are induced in monocytes from CHB patients when stimulated with TLR8 agonists.

### TLR8 Agonism Induces T_FH_ Cells Differentiation

**Figure 2:**
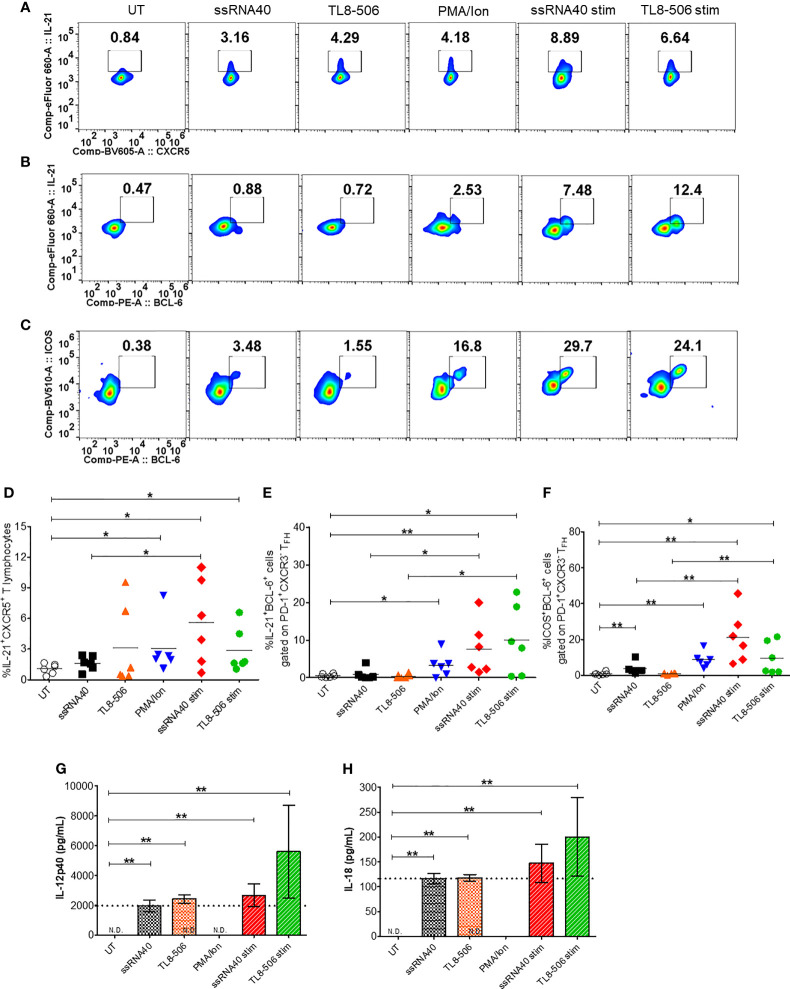
TLR8 agonism induces T_FH_ differentiation. PBMCs from CHB patients (n=6) were left untreated (UT) or treated with TLR8 agonists ssRNA40 or TL8-506 for 5 days. ssRNA40 stim and TL8-506 stim indicates subtraction of overnight PMA/Ion stimulations from the respective values of TLR8 + PMA/Ion re-stimulations. Illustrative flow plots show **(A)** intracellular expression of T_FH_ signature cytokine IL-21 gated on cT_FH_ (CD4^+^CXCR5^+^CD3^+^ T lymphocytes) cells, co-expression of GC-like T_FH_ development factors **(B)** IL-21^+^BCL-6^+^ and **(C)** ICOS^+^BCL-6^+^ gated on PD-1^+^CXCR3^-^ T_FH_ cells upon treatment with TLR8-specific ligands. Frequencies are shown for **(D)** IL-21^+^CXCR5^+^, **(E)** IL-21^+^BCL-6^+^ and **(F)** ICOS^+^BCL-6^+^ cells. Pro-inflammatory cytokines secreted in the supernatants collected from indicated conditions of PBMC cultures quantified by Luminex multiplex immunoassay. Bar graphs depict mean levels of cytokines **(G)** IL-12p40 (pg/mL) and **(H)** IL-18 (pg/mL) upregulated by TLR8-specific agonists. Dotted line indicates comparison between TLR8 stimulations. The differences between stimulations were evaluated by one-way ANOVA or Kruskal-Wallis test for multiple comparisons. P values ≤0.05*, 0.01** indicate statistical significance levels. N.D., not detected; cT_FH,_ circulating follicular helper T cells; GC-like T_FH,_ germinal center-like T_FH_; PMA, phorbol-12-myristat-13-acetate; Ion, ionomycin; Stim, stimulation.

Having shown potent induction of T_FH_ polarizing cytokine IL-12p40 (12) in monocytes from CHB samples, we next examined the impact of TLR8 agonism on T_FH_ phenotypes. Circulating CD4^+^CXCR5^+^ cells in peripheral blood share characteristics and functional phenotypes with GC-like T_FH_ cells (20). The expression of signature cytokine IL-21, BCL-6 (a master regulator of T_FH_ cells programing and differentiation) and CD40 ligand (CD40L) in T_FH_ cells provide signals to B cells that are required for Ig class-switching recombination and high affinity antibody productions (21). The expression of inducible co-stimulatory molecule (ICOS) indicates T_FH_ development, migration and their active state (22). We asked whether TLR8-induced cytokines modulate these immunophenotypic profiles of circulating T_FH_ cells (cT_FH_). CHB patient samples were stimulated with TLR8-specific agonists ssRNA40 or TL8-506 in the presence or absence of mitogenic stimulation using PMA/Ion (required for induction of BCL-6 and ICOS). The sequential gating strategy for T_FH_ cell panel is presented in **Supplementary Figure 2A**. Flow plots (**Figure 2A**) show that the frequency of IL-21 producing cT_FH_ cells (IL-21^+^CXCR5^+^) gated on CD4^+^ T cells was significantly increased in conditions with ssRNA40 or TL8-506. The data represented for ssRNA40 stim or TL8-506 stim are response found in TLR8 agoniost+PMA/Ion re-stimulated response subtracted with PMA/Ion alone response (**Figures 2A–F**). The co-expression of GC-like T_FH_ markers IL-21^+^BCL-6^+^ and ICOS^+^BCL-6^+^ were significantly increased in ssRNA40 stim or TL8-506 stim conditions (**Figures 2B–F**). Since proinflammatory cytokines IL-12p40 and IL-18 were induced in monocytes in response to TLR8 agonism (**Figure 1**), we measured levels of these cytokines in the PBMC culture supernatants. As expected, cultures incubated with TLR8-specific agonists ssRNA40 or TL8-506 markedly induced the secretion of both these cytokines (**Figures 2G, H**).

Next, we asked whether TLR8-specific signaling modulates subsets of circulating T_FH_ cells in CHB patients. When compared with untreated negative control, PBMCs in conditions with ssRNA40 or TL8-506 had significantly increased frequencies of cT_FH_ subsets cT_FH_1 (CXCR3^+^CCR6^-^) and T_FH_1/17 (CXCR3^+^CCR6^+^) (**Supplementary Figures 2B, D, G**), while no differences were observed for global cT_FH_ (CXCR5^+^CD4^+^ gated on CD3^+^ T lymphocytes) cells, subsets cT_FH_2 (CXCR3^-^CCR6^-^) and cT_FH_17 (CXCR3^-^CCR6^+^) (**Supplementary Figures 2C, E, F**). It has been previously reported that cT_FH_1 and cT_FH_1/17 are the source for IL-21 and IFNγ, whereas cT_FH_2 and cT_FH_17 are source for anti-inflammatory cytokines IL-4, IL-10 and IL-17A. These results collectively suggest that TLR8-specific signaling induces IL-21 producing T_FH_ subsets in the setting of CHB.

### Selgantolimod Treatment Enhanced T_FH_ Differentiation and Rescued Defective Antigen-Specific Activation of T_FH_ Cells

After *in vitro* demonstration of T_FH_ differentiation with TLR8 agonism, we tested samples from CHB patients given a single oral dose of Selgantolimod. Surprisingly, in post-Selgantolimod treated samples that had been obtained only 8 hours after dosing, a significant induction of GC-like signature T_FH_ factors IL-21^+^BCL-6^+^, ICOS^+^BCL-6^+^ and ICOS^+^CD40L^+^ cells were observed (**Supplementary Figures 3A–H**).

**Figure 3:**
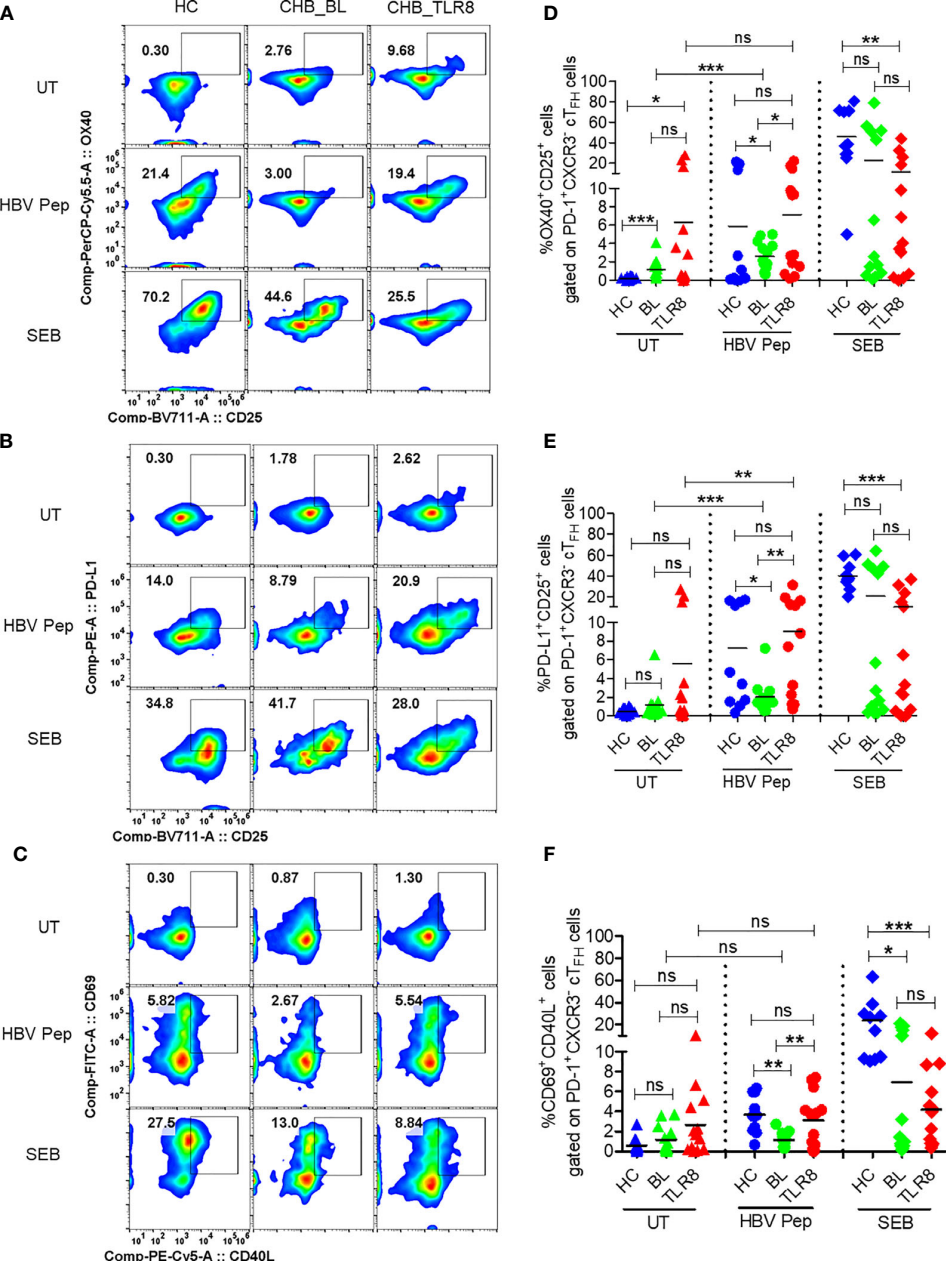
Selgantolimod treatment rescued defective antigen-specific activation of T_FH_ cells. PBMCs isolated from HBV vaccinated healthy controls (HC, n=10) and paired samples from CHB patients at baseline (BL) or 8h post-TLR8 agonist (single oral dose of 1.5-3 mg/Kg, Selgantolimod) administration (CHB_TLR8) (n=10-14) were stimulated with combination of HBsAg and HBV peptides pool for 18-24h. Staphylococcal enterotoxin B (SEB, T cell antigen receptor stimulus) and AIM-V medium alone (UT) served as positive and negative controls, respectively. Flow cytometry analysis was performed using AIM assay panel antibodies (**Supplementary Table 1**). Flow plots represent co-expression of HBV-specific activation induced T_FH_ markers (AIM). Representative pseudo color plots show comparative co-expression of **(A)** OX40^+^CD25^+^, **(B)** PD-L1^+^CD25^+^ and **(C)** CD69^+^CD40L^+^ cells gated on GC-like T_FH_ (PD-1^+^ CXCR3^-^ cT_FH_) cells in HC and CHB (BL and TLR8) samples. Numbers shown inside the flow box indicate percentage of events. Relative frequencies of GC-like T_FH_ markers shown in graph as percentage of cells expressing **(D)** OX40^+^CD25^+^, **(E)** PD-L1^+^CD25^+^ and **(F)** CD69^+^CD40L^+^. Each symbol in the graph shows individual donors and the dotted line distinguishes the data between stimulations. A two-tailed unpaired or paired, non-parametric or the Wilcoxon signed-rank Student’s t test were conducted to evaluate the differences between HC, BL and 8h post-TLR8 treated samples. P values of ≤0.05*, 0.01**, 0.001*** indicate statistical significance. HC, vaccinated healthy control; CHB, chronic hepatitis B; HBV Pep, HBsAg/HBV peptides pool; ns, no significance.

Next, we employed a cytokine-independent method for detection of HBsAg-specific T_FH_ cell activation by measuring the upregulation of activation induced markers (AIM) CD69, CD25, OX40, PD-L1 and CD40L (16, 17). Both frequencies of CD3^+^CD4^+^CXCR5^+^CXCR3^-^PD-1^+^ T_FH_ and their activation, indicated by surface expression of OX40^+^CD25^+^, PD-L1^+^CD25^+^ and CD69^+^CD40L^+^, was increased in vaccinated individuals upon *in vitro* stimulation with HBV antigens or SEB compared to the negative control (**Figures 3A–F**, **Supplementary Figure 4**). Expectedly, baseline samples from CHB patients displayed low HBV specific activation of T_FH_ indicating defective response. Importantly, a significant increase in AIM response was evident in 8 hour samples, which was comparable to HBV vaccinated samples (**Figures 3A–F**). Additionally, the frequencies of PD-1^+^CXCR3^-^ cT_FH_ cells, but not total CXCR5^+^ cT_FH_ cells, were significantly higher in samples collected at post-TLR8 time-point (**Supplementary Figures 4B, C**). These data demonstrate that TLR8-specific signaling can normalize defective antigen specific T_FH_ response.

### TLR8 Signaling Increased Memory B, Plasma B, and Modulated B Cell Subsets

**Figure 4:**
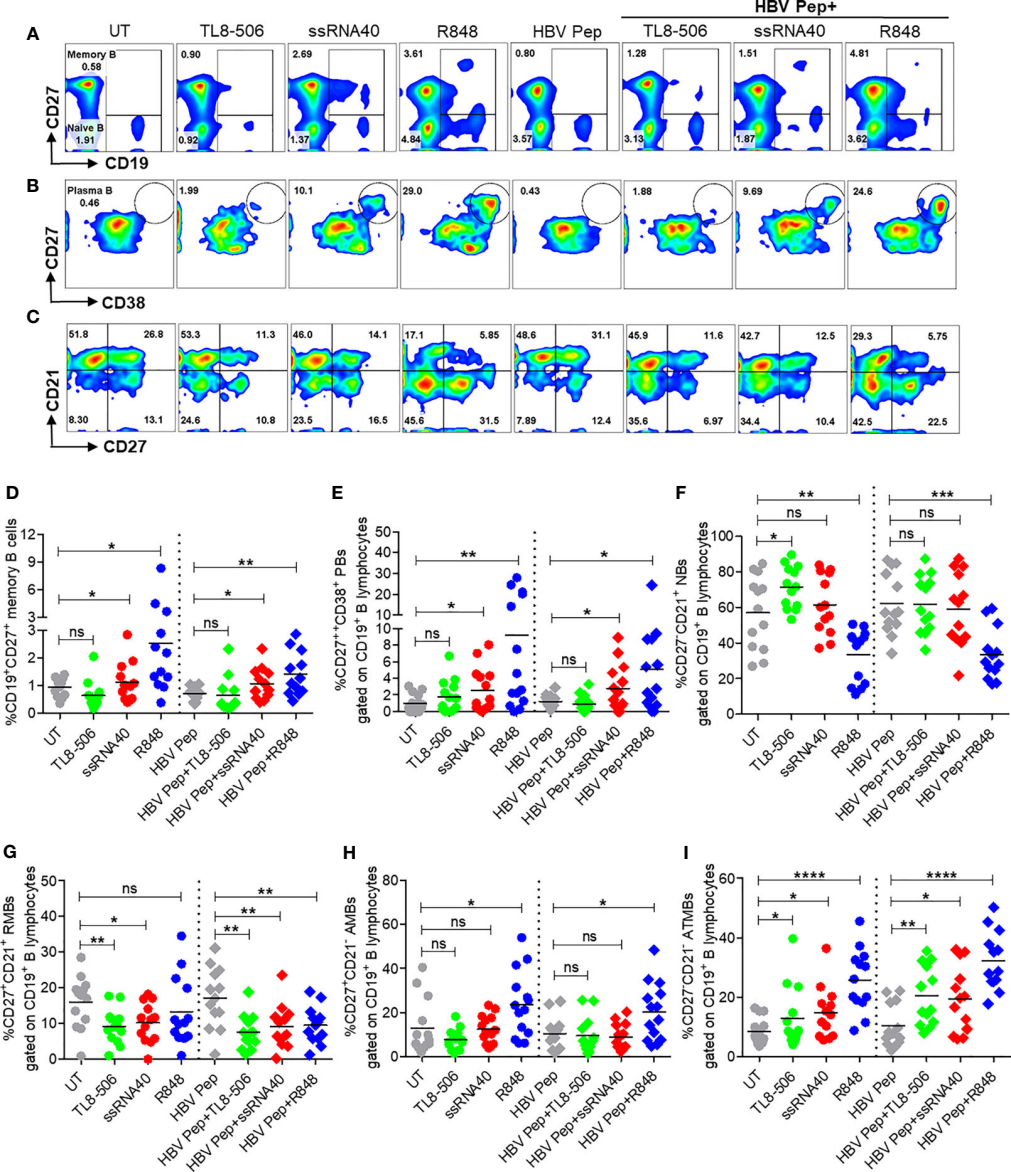
Activation of TLR8 pathway increased memory B, plasma B, and modulated B cell subsets. PBMCs collected from CHB infected patients (n=10-14), were left untreated (UT) or treated with specific TLR8 ligands TL8-506, ssRNA40 or TLR7/8 ligand R848 along with or without HBsAg and HBV Pep and cultured for 5 days. Flow cytometry evaluation of B cell subsets at the end of culture for **(A)** MB (CD27^+^), **(B)** plasma B (CD27^++^CD38^+^) and **(C)** B cell subsets defined by CD27 and CD21 expressions, gated on CD19^+^ B lymphocytes. Dot plots show the frequencies of **(D)** MB (CD19^+^CD27^+^), **(E)** plasma B (CD19^+^CD27^++^CD38^+^), **(F)** NBs (CD19^+^CD27^-^CD21^+^), **(G)** RMBs (CD19^+^CD27^+^CD21^+^), **(H)** AMBs (CD19^+^CD27^+^CD21^-^) and **(I)** ATMBs (CD19^+^CD27^-^CD21^-^). Numbers inside the quadrants represent percentage of subsets. Dotted lines distinguish TLR8 without or with HBV Pep treatment conditions. Statistical significance determined by two-tailed, non-parametric or the Wilcoxon signed-rank Student’s t test for comparison between stimulations. P values ≤0.05*, 0.01**, 0.001***, 0.0001**** indicate statistical significance. ns, no significance; MB, memory B; PB, plasma B; NBs, naïve B; RMBs, resting memory B; AMBs, activated memory B; ATMBs, atypical memory B cells.

To determine whether the activation of TLR8 pathway and resulting T_FH_ differentiation impacts B cell populations in CHB infection, frequencies of various B cell subsets were examined after incubation of PBMC with ssRNA40, TL8-506 or R848 in presence or absence of HBV antigens for 5 days. Frequencies of CD19^+^ B cells did not change with these treatments (**Supplementary Figures 5A–C**). Activation of TLR8 pathway with ssRNA40 or of TLR7/8 with R848 significantly expanded the frequencies of memory B cells (CD19^+^CD27^+^), plasma B cells (CD19^+^CD27^++^CD38^+^), plasmablasts (CD19^+^CD27^++^CD38^+^CD138^-^) and plasma cells (CD19^-^CD138^+^) (**Figures 4A–E** and **Supplementary Figures 5D, E**). While TL8-506 treatment did not result in significant changes in frequencies of these populations, it increased naïve B (CD19^+^CD27^-^CD21^+^) and long-lived plasma (CD19^-^CD138^+^) cells (**Figure 4F** and **Supplementary Figure 5D**). Addition of HBV antigens in addition to TLR8 agonism didn’t alter naïve B (CD19^+^CD27^-^CD21^+^), resting memory B (CD19^+^CD27^+^CD21^+^) or activated memory B cells CD19^+^CD27^+^CD21^-^) (**Figures 4A–I** and **Supplementary Figures 5D, E**).

Previously we reported that atypical memory B cells that expressed high levels of FcRL family receptors were expanded in CHB patients and this sub-population lacks HBsAg-specific B cell response (6). Here we observed that TLR8 stimulation expanded memory B cells with an atypical phenotypic signature (CD19^+^CD21^-^CD27^-^) (**Figure 4C, I**). We believe that this subset lacking CD21 and CD27 that is expanded in response to TLR7/8 stimulation does not represent an elevation of dysfunctional cells. R848 is typically used to increase memory B cells for measuring B cell responses with ELISPOTs for chronic HBV infection (9, 23). It is also known that during malaria, B cells with similar ‘atypical’ phenotype are in fact functional and produce neutralizing antibodies (24).

### TLR8 Signaling Promotes IL-12-Dependent GC-Like T_FH_ Cell Differentiation and Improved IL-21 Production

**Figure 5:**
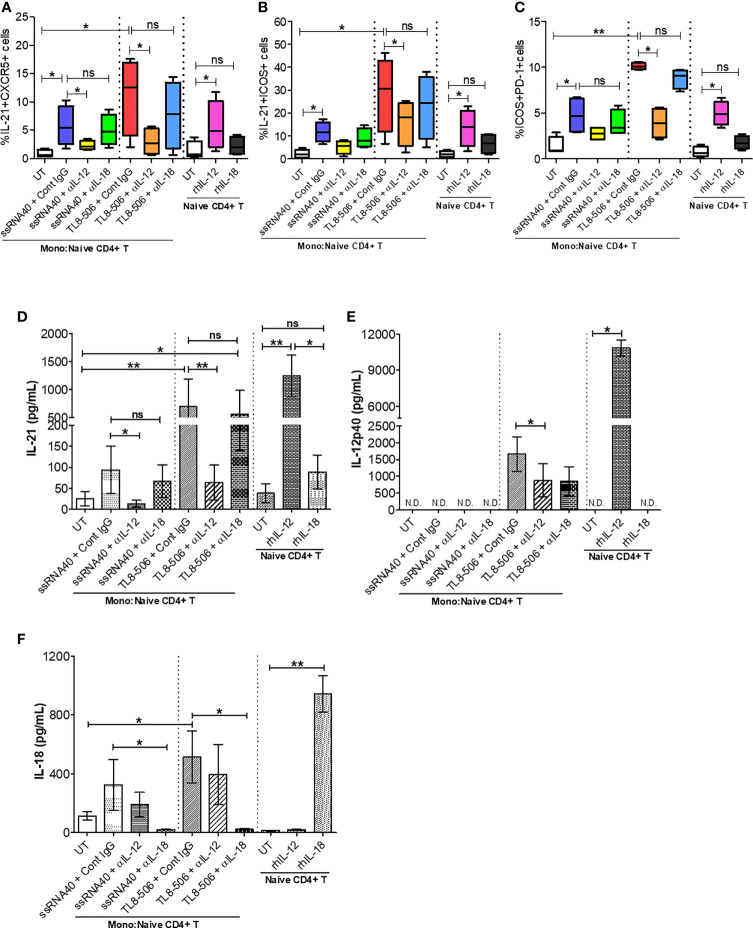
TLR8 signaling promotes IL-12-dependent GC-like T_FH_ cell differentiation and improved IL-21 production. Autologous naïve CD4^+^ T cells were isolated from PBMCs of HC (n=2) and CHB patients (n=2) and co-cultured with CD14^+^ enriched monocytes (as APCs, 2:1 ratio) previously stimulated with medium (UT, negative control), TLR8-specific ligands ssRNA40 or TL8-506 along with isotype control IgG, anti-IL-12 or anti-IL-18 neutralizing (blocking) Ab and cultured for 6 days in the presence of SEB followed by re-stimulation with PMA/Ion to activate T_FH_ cells. Flow cytometry was carried out and the frequencies of cells co-expressing T_FH_ markers are shown for **(A)** IL-21^+^CXCR5^+^, **(B)** IL-21^+^ICOS^+^ and **(C)** ICOS^+^PD-1^+^. Cytokine levels were quantified in the supernatants collected from monocyte-naïve CD4^+^ T co-cultures as well as naïve CD4^+^ T cell culture by Luminex multiplex immunoassay. Bar graph depicts the levels of **(D)** IL-21 (pg/mL), **(E)** IL-12p40 (pg/mL) and **(F)** IL-18 (pg/mL) secretion in response to TLR8-specific agonists and blocked by anti-IL-12, anti-IL-18 neutralizing Abs or upon incubation with human recombinant proteins IL-12 or IL-18. Statistical significance calculated by one-way ANOVA or Kruskal-Wallis test for comparison between stimulation conditions. P values ≤0.05*, 0.01** considered significant, ns, no significance; APCs, antigen-presenting cells; SEB, staphylococcal enterotoxin B; rhIL-12/IL-18, recombinant human protein IL-12/IL-18; Cont. IgG, isotype control IgG; N.D., not detected.

In order to discern the mechanism behind TLR8-mediated differentiation and proliferation of T_FH_ cells, we performed co-culture experiments of isolated monocytes and CD4^+^ T cells. CD14^+^ monocytes were treated with ssRNA40 or TL8-506, in presence of isotype control IgG or anti-human IL-12 or anti-human IL-18 neutralizing (blocking) antibodies followed by co-culture with autologous naïve CD4^+^ T cells for 6 days. Monocytes stimulated with TLR8-specific agonists induced T_FH_ cell markers on CD4^+^ T cells (CD4^+^CXCR5^+^IL-21^+^, IL-21^+^ICOS^+^ and ICOS^+^PD-1^+^) in samples from healthy individuals and CHB patients. Untreated monocytes co-cultured with autologous naïve CD4^+^ T cells failed to induce IL-21 and other differentiation factors (**Figures 5A–C** and **Supplementary Figures 6A–C**).

In humans, TLR8 pathway-specific secretion of proinflammatory cytokine IL-12 sends the signal responsible for IL-21 production and drives the T_FH_ cell differentiation (12, 25). In CHB patients, T_FH_ cell frequencies are high due to hyperactivation and these cells lack IL-21 cytokine production. We therefore explored whether TLR8-specific cytokine induction will promote circulating T_FH_ cell differentiation and recover defective IL-21 production in PBMCs of CHB patients. IL-12p40 and IL-18 secretion in monocytes and IL-21 production from newly generated T_FH_ cells were quantitatively analyzed in supernatants of co-culture experiments and naïve CD4^+^ T cell culture by Luminex multiplex immunoassay. As expected, IL-12 neutralization (ssRNA40+α-IL-12 and more clearly TL8-506+α-IL-12) in CD14^+^ monocytes-naïve CD4^+^ T cells co-culture significantly decreased IL-21 production as well as the co-expression of GC-like T_FH_ markers IL-21^+^ICOS^+^ and ICOS^+^PD-1^+^, on cT_FH_ cells (CD3^+^CD4^+^CXCR5^+^); IL-18 neutralization (ssRNA40+α-IL-18 or TL8-506+α-IL-18) did not have similar effect (**Figures 5A–C** and **Supplementary Figures 6A–C**). Correspondingly, the secretion of cytokine IL-21 was significantly reduced in co-cultures pre-treated with IL-12 neutralizing antibody (ssRNA40+α-IL-12 and more notably with TL8-506+α-IL-12). IL-18 blocking again did not have an effect on IL-21 production in either ssRNA40+α-IL-18 or TL8-506+α-IL-18 antibody treatment conditions (**Figures 5D–F**). IL-18 production remained intact with α-IL-12 treatments. To further justify that TLR8-specific IL-12 cytokine-dependent signal is responsible for T_FH_ cell differentiation and IL-21 cytokine production, we tested naïve CD4+ T cells isolated from PBMCs of HC and CHB patients that were left untreated or directly incubated with recombinant human IL-12 or IL-18 and cultured for 5 days in the absence of CD14^+^ monocytes. The frequency of IL-21 producing T_FH_ cells was significantly increased when incubated with rhIL-12, but not with rhIL-18 (**Figures 5A–F** and **Supplementary Figure 6F**). It is important to note here that effect of TLR8 was not specific to CXCR5^+^ T_FH_ and the agonist activated CD4^+^ T cells including CXCR5^-^ cells in an IL-12 dependent manner (**Supplementary Figures 6D, E**). Taken together, these data demonstrate that TLR8-specific IL-12-induction in monocytes plays a critical role in the T_FH_ cells differentiation, which can restore the deficient IL-21 production in CHB.

### T_FH_ Cells Differentiated by TLR8 Agonists Support The Generation of Memory B and Plasma B Cells

**Figure 6:**
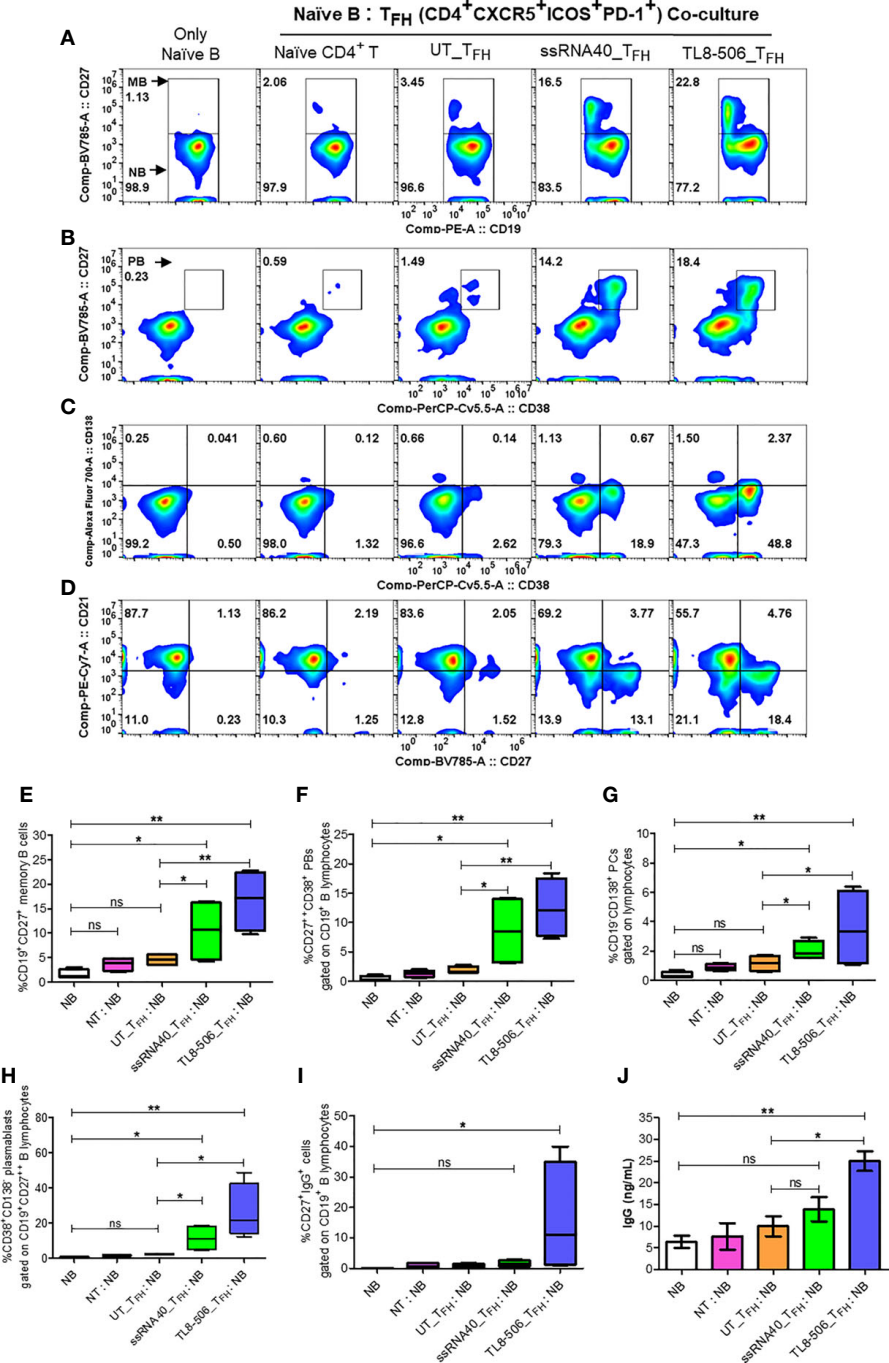
T_FH_ cells differentiated by TLR8 pathway support the generation of memory B and plasma B cells. GC-like T_FH_ cells (PD-1^+^CXCR3^-^ cT_FH_) differentiated with TLR8 agonists were sorted and co-cultured with autologous naïve B cells from healthy donors (n=2) and CHB patients (n=2) at T_FH_:naïve B ratio of 1:2 in the presence of staphylococcal enterotoxin and generated B cell subsets and IgG examined. Flow cytometry analysis carried out using B cell panel antibodies show pseudo color flow plot of **(A)** Memory B (CD27^+^), **(B)** Plasma B (CD27^++^CD38^+^), **(C)** PCs (CD27^++^CD38^-^CD138^+^), PBs (CD27^++^CD38^+^CD138^-^) and **(D)** B cell subsets NBs (CD27^-^CD21^+^), RMBs (CD27^+^CD21^+^), AMBs (CD27^+^CD21^-^), ATMBs (CD27^-^CD21^-^), gated on CD19^+^ B lymphocytes. Numbers inside the quadrant represents the percentage of events. The frequencies of different B cell subsets are presented in the bar graphs **(E–H)**, as indicated. **(I)** frequencies of IgG^+^ cells, gated on CD19^+^CD27^+^ memory B cells. **(J)** IgG levels (ng/mL) secreted by memory B cells in the supernatants recovered from naïve B-T_FH_ co-culture experiments detected by high sensitivity ELISA assay. Statistical analyses done by one-way ANOVA or Kruskal-Wallis test for comparison between cell culture conditions. P values ≤0.05*, 0.01** represent statistical significance. NBs, naïve B; NT, Naïve CD4^+^ T; PCs, plasma cells; PBs, plasmablasts; RMBs, resting memory B; AMBs, activated memory B; ATMBs, atypical memory B cells; ns, no significance.

To evaluate the utility of newly generated T_FH_ cells as true helpers of B cells, we sorted CD4^+^CXCR5^+^CXCR3^-^PD-1^+^ICOS^+^ T_FH_ generated with UT, ssRNA40 or TL8-506 pre-exposed CD14^+^ monocytes and assessed their ability to promote plasma B cell differentiation after co-culture with autologous naïve B cells for 7 days. T_FH_ generated from TLR8 primed monocytes enhanced the differentiation of global memory B (CD27^+^), plasma B (CD27^++^CD38^+^), long-lived antibody-producing plasma cells (CD27^++^CD38^-^CD138^+^) and plasmablasts (PBs) (CD27^++^CD38^+^CD138^-^), whereas T_FH_ derived from UT or naïve CD4^+^ T cells co-cultured with B cells failed to induce memory, plasma B and long-live plasma cell differentiation (**Figures 6A–J**).

Additionally, significantly higher frequencies of IgG^+^ (CD19^+^CD27^+^) memory B cells and robust IgG production in TL8-506 primed T_FH_-naïve B co-culture condition compared to naïve B without or with naïve CD4^+^ T or UT_T_FH_ co-culture conditions was present (**Figures 6I, J**). Flow plot in **Figure 6D** shows ssRNA40 and TL8-506-primed T_FH_-naïve B co-culture conditions resulted in a reduction in naïve B (CD19^+^CD21^+^CD27^-^) and increase in resting memory B (CD19^+^CD21^+^CD27^+^), activated memory B (CD19^+^CD21^-^CD27^+^) and atypical memory B (CD19^+^CD21^-^CD27^-^) cell populations, relative to non-TLR8 conditions. Thus T_FH_ differentiated with TLR8-treatment support the generation of memory B cells and their subsets in CHB samples.

### Selgantolimod Treatment Enhanced B Cell Responses

**Figure 7:**
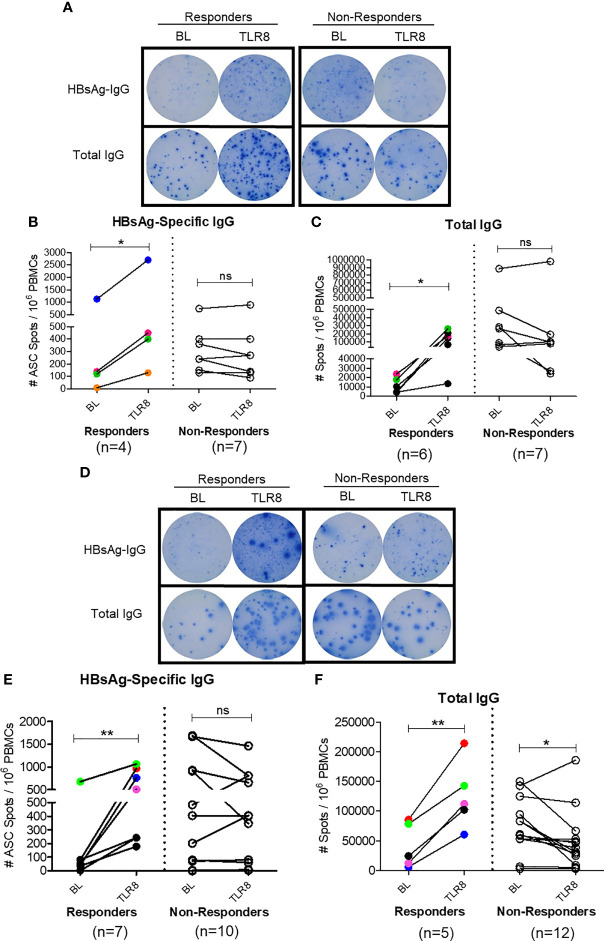
Selgantolimod treatment enhanced B cell responses. PBMCs from BL or Selgantolimod (TLR8) administered HC (phase 1a **A–C**) and CHB (phase 1b, **D–F**) samples were stimulated as detailed in methods to effectively induce memory B cells proliferation. **(A, D)** Representative ELISPOT results show IgG spots in responders and non-responders, **(B, E)** HBsAg-specific IgG and **(C, F)** total IgG secreting B cells from phase 1a and phase 1b samples. Statistical significance calculated by two-tailed paired Student’s t test or the Wilcoxon signed-rank. Color-coded symbols in responders group indicate same clinical sample tested for HBs-IgG and total IgG. Responder defined as subject with >=1.5-fold improvement in SFU in TLR8 compared with BL time-point. P value of ≤0.05*, 0.01** indicate statistical significance. ns, no significance. BL- baseline; TLR8- toll-like receptor 8; IgG, immunoglobulin; n, number of paired samples.

To assess whether this mechanism of enhancing T_FH_ response impacts B cell response *in vivo*, we next examined the effect of Selgantolimod oral treatment in paired samples from before and 8 hours after treatment. For this experiment, in addition to phase 1b clinical samples, clinical samples from phase 1a trial in CHB negative healthy subjects with known positive HBV vaccination status were tested for HBsAg-specific IgG and total IgG with ELISPOT assays. We defined responders as those who exhibited an increase of ≥1.5-fold SFU/ml after Selgantolimod administration. Using this as criteria, there were 4/11 responders for HBs specific and 6/13 responders for total IgG response among phase 1a samples (**Figures 7A–C**). In samples from CHB patients (phase 1b), HBs specific IgG spots increased in 6/17 subjects and total IgG in 5/18 subjects (**Figures 7D–F**). Most subjects that showed improvement in spots had lower baseline IgG response, though there was no clear cutoff for determining responders. These outcomes suggest that TLR8 treatment has potential to augment memory B cell response in a subset of CHB subjects. It will be important to identify characteristics of responders or non-responders; however, here we did not see positive correlation between T_FH_ response and ELISPOT response in the small number of responders.

## Discussion

In this investigation we demonstrated that triggering of TLR8 induces IL-12 production from monocytes, which in turn leads to differentiation of CD4^+^ T cells into IL-21 producing T_FH_ in peripheral blood samples from CHB patients. Accordingly, co-culture of these differentiated T_FH_ with autologous B cells resulted in B cell differentiation into plasma cells and promoted IgG production. Finally, in a fraction of CHB patients treated with a selective TLR8 agonist, improved HBsAg-specific T_FH_ and B cell responses were observed. Therefore, our study established that TLR8 signaling has potential to restore a critical defect in T-B interaction that is necessary for robust B cell response.

Humoral immunity requires interaction between B cells and specialized populations of CD4^+^ T cells, the CD4^+^CXCR5^+^ follicular helper (T_FH_) cells, the latter help generate memory B cells and long-lived plasma cells (26); both these immune cell types are dysfunctional in CHB patients. Since HBcAg-specific antibodies are generated during infection, this defect is selective to HBsAg-specific cells, potentially due to high levels of circulating HBsAg present in patients (27). Indeed, HBs specific but not HBc specific B cells show characteristics of impaired cells characterized by atypical memory phenotype and poor differentiation into antibody secreting cells (5, 6, 28, 29). At the same time, T_FH_ have dysregulated response to HBsAg that associates with HBV persistence (30). Our results also demonstrate that HBsAg-specific T_FH_ response is defective in CHB patients relative to HBV vaccinated controls. We show here that IL-12 induction by monocytes in response to TLR8 signaling was able to rescue these defective T_FH_. Specifically, signaling through TLR8 resulted in differentiation of T_FH_ into phenotypes consistent with bona fide helpers of B cells i.e., PD-1^+^BCL-6^+^ICOS^+^IL-21^+^, in samples from CHB patients. *In vitro* use of Selgantolimod resulted in a similar increase in frequency of circulating PD-1^+^ T_FH_ in another study (31). We have only studied peripheral T_FH_ here due to limitations in access to lymphoid cells from patients. As such this may not entirely reflect the impact on antibody generation process, which occurs in secondary lymphoid organs, specifically in structures called germinal centers. However, specific subsets of circulating T_FH_ are identified which share similar phenotypes, transcriptional and functional characteristics with germinal center T_FH_ cells (20); this peripheral population allows an easily accessible means to examine phenotypic and functional state of these cells during diseases and to investigate effects of interventions on them (21). Canonical T_FH_ differentiation starts with dendritic cell priming of naïve CD4^+^ T cell whereupon receiving cytokine signals, expression of CXCR5 on CD4^+^ T cell allows early T_FH_ to migrate to T-B cell border and undergo further differentiation. Among DC derived cytokines, IL-12 and TGF-β1 are most efficient at inducing human naïve CD4^+^ T cells to express T_FH_ molecules CXCR5, ICOS, IL-21 and BCL-6 (32–36). Recently, it was discovered that TLR8 signaling results in differentiation of functional T_FH_; the intact ssRNA in attenuated vaccines triggers TLR8 on monocytes, inducing IL-12 production, which in turn differentiates naïve CD4^+^ T cells into functional T_FH_. These T_FH_ cells support plasma cell generation and IgG production from B cells (12).

CHB infection is associated with significant immune dysregulation, whether TLR8 agonism will lead to TFH differentiation and subsequent B cell help was therefore not a given. Reports have shown both functional (10) and defective TLR8 response (37) in CHB. However, *in vitro* testing of Selgantolimod showed comparable levels of cytokines in PBMC from healthy and CHB subjects (31). In clinical trial of this agonist in healthy subjects, we previously showed IL-12p40, IL-12p70, IL-1RA and IL-18 induction in serum (14), here we show T_FH_ polarizing cytokines, IL-12 is induced with the agonist in monocytes from CHB patients which led to naïve CD4^+^ T cells to differentiated into CXCR5^+^BCL-6^+^ICOS^+^IL-21^+^ T_FH_. A significant finding here is demonstration of HBsAg-specific T_FH_ response (ICOS^+^BCL-6^+^ T_FH_ and upregulation of AIM markers) in CHB patients treated with a single dose of Selgantolimod. IL-12p40 and IL-12-p70 are induced within 4 hours after oral administration of this agonist (14); we hypothesize that IL-12p70 provided requisite signal to naïve CD4^+^ T cells in treated patients, which was manifested by the expression of T_FH_-specific growth factors when PBMC were tested in our *in vitro* culture assays. Mechanistically, TLR8 agonism induced T_FH_ with phenotypes bona fide helpers which aided plasma cell generation and IgG response. In our study this was reflected in results from naïve B-T_FH_ co-culture and B cell ELISPOT assays, which demonstrated enhanced total IgG and HBsAg-specific B cell responses after TLR8 agonist treatment. It may seem surprising that an improvement in B cell response was present in an acute treatment setting (8 hours) with a single dose of Selgantolimod. However, it is important to note that these B cell responses were not measured *ex vivo*, rather the ELISPOT assay measures memory B cell responses generated after short-term cultures of 5 days. We believe that Selgantolimod treatment generated favorable conditions, such as effective T_FH_ response and cytokine IL-21, which aided these improved B cell responses *in vitro*.

These finding have significance for use of TLR8 agonism for hepatotropic HBV; liver derived cells respond to TLR8 agonism by inducing IL-12 and IL-18 (10). In a woodchuck model of chronic HBV, this same TLR8 agonist resulted in sustained antiviral response and loss of HBs antigen (38). This model uses a surrogate virus and the translatability of these findings to CHB in humans is uncertain, however, the same agonist resulted in similar induction of cytokines IL-12p40, IL-12p70, IL-1β, IL-6, IL-18 and TNF-α *in vivo* in humans (14, 13, 38) and in cultures (here). In a separate study, IL-12, IL-1β and IFN-β induced by ssRNA were shown critical for T_FH_ differentiation, this effect was mediated by TLR signaling adaptor TRIF (39).

There are certain limitations of our study. For more precise evaluation of antigen specific T_FH_ and B cells, use of class II tetramers and HBsAg specific B cell probes, respectively, is needed. It is out of the scope for this investigation due to limited availability of frozen PBMC from these previously completed clinical trials for further experiments. It is also important to show whether an increase in the IgG secreting B cells (ASCs) observed in ELISPOT from some patients correlates with enhanced monocytic IL-12 production or with expression of GC-like T_FH_ growth factors (IL-21^+^BCL-6^+^, IL-21^+^CD40L^+^ and ICOS^+^BCL-6^+^) or B cell phenotypes. However, due to small sample size it was not possible to perform such correlation analyses in ‘responders’ and ‘non-responders’ to TLR8 agonism. A focus on peripheral examination of T-B responses with lack of any investigation into tissue or lymph node responses is another limitation here. T_FH_-B cell responses will be studied in the ongoing Phase 2 clinical trial, which could establish the true nature of T_FH_-B cell interactions in restoring clinically relevant anti-HBs response in CHB patients.

Our data has significance for our understanding of impaired HBV-specific protective immunity in CHB patients. Strategies to achieve a functional cure are focused on restoring HBs-antibody responses in CHB patients. HBs antigen-based vaccine candidates have shown mixed results when tested in clinical trials in chronically infected patients (40). Our data support further testing of TLR8 agonism in HBV functional cure approaches that include a combination of antiviral and immune modulatory agents.

## Data Availability Statement

The original data presented in the study are included in the article and **Supplementary Material**. Further inquiries can be directed to the corresponding author.

## Ethics Statement 

The studies involving human participants were reviewed and approved by University of Maryland, Baltimore. The patients/participants provided their written informed consent to participate in this study.

## Author Contributions 

NA designed methods, performed the experiments, analyzed data and prepared results. LT provided clinical expertise and provided CHB samples. SKT, DC, JW, and SF provided samples from Selgantolimod clinical trials and critically edited the manuscript. SK participated in study design, critically edited the manuscript and provided resources. BP designed the study, analyzed and interpreted the results, wrote the manuscript and provided funding. All authors contributed to the article and approved the submitted version.

## Funding

The study was financially supported by Gilead Sciences, Foster City, CA and IHV, University of Maryland school of Medicine, Baltimore, MD.

## Conflict of Interest

SKT, DC, JJW and SPF were employed by Gilead Sciences Inc. BP received financial support paid to University of Maryland from Gilead Sciences. The authors also declare that this study received funding from Gilead Sciences. The funder had the following involvement with the study: interpretation of data/editing of manuscript.

## Publisher’s Note

All claims expressed in this article are solely those of the authors and do not necessarily represent those of their affiliated organizations, or those of the publisher, the editors and the reviewers. Any product that may be evaluated in this article, or claim that may be made by its manufacturer, is not guaranteed or endorsed by the publisher.

## Abbreviations

CHB, chronic hepatitis B; HBV, hepatitis B virus; T_FH_, follicular helper T cells; cT_FH_, circulating follicular helper T cells; PBMCs, peripheral blood mononuclear cells; APCs, antigen-presenting cells; TLR8, toll-like receptor 8; HC, healthy control; GC, germinal center; HBsAg, hepatitis B surface antigen; ssRNA40, single stranded RNA40; mg, milligram; Kg, kilogram; PBS, phosphate-buffered saline, FBS, fetal bovine serum; AIM assay, activation induced marker assay; SEB, staphylococcal enterotoxin B; ELISPOT, enzyme-linked immune absorbent spot; ASC, antigen-specific antibody secreting cell; IL, interleukin; LPS, lipopolysaccharide; IMQ, imiquimod; TNF-α, tumor-necrosis factor alpha; MB, memory B; PB, plasma B; PCs, plasma cells; PBs, plasmablasts; NBs, naïve B; RMBs, resting memory B; AMBs, activated memory B; ATMBs, atypical memory B; PMA, phorbol-12-myristat-13-acetate; Ion, ionomycin; LEP, large envelop protein; Stim- stimulation; FACS, fluorescence-activated cell sorting; ICS, intracellular staining; UT, untreated; BL, baseline; IgG, immunoglobulin G; ELISA, enzyme-linked immunosorbent assay; HBV Pep; hepatitis B virus peptide; ANOVA, analysis of variance.
