# Supplementary material for: Follicular Helper T (TFH) Cell Targeting by TLR8 Signaling For Improving HBsAg-Specific B Cell Response In Chronic Hepatitis B Patients
Source: Front Immunol. 2021 Aug 26;12:735913. doi: 10.3389/fimmu.2021.735913 (PMC8428528; doi:10.3389/fimmu.2021.735913)

Supplementary Table 1. Panels of flow cytometry antibodies

Panels	Sl. No.	Fluorochrome	Markers	Clone	Company	Cat. #
**T_{FH}**	1	BV421	PD-1 (CD279)	EH12.2H7	Biolegend	329920
	2	BV510	ICOS	C398.4A	Biolegend	313525
	3	BV570	CD4	RPA-T4	Biolegend	317445
	4	BV605	CXCR5	J252D4	Biolegend	356930
	5	BV711	CD25	M-A251	Biolegend	356137
	6	BV785	CCR6	G034E3	Biolegend	353421
	7	FITC	CD69	FN50	Biolegend	310904
	8	PE	BCL6	K112-91	BD Pharmigen	561522
	9	PE-Dazzle 594	CD3	UCHT1	Biolegend	300450
	10	PE-Cy5	CD40L	24-31	Biolegend	310808
	11	PerCP-Cy5.5	OX40/CD134	Ber-ACT35	Biolegend	350009
	12	PE-Cy7	CXCR3	G025H7	Biolegend	353719
	13	eFluor 660	IL-21	eBio3A3-N2	Invirogen	50-7219-42
	14	AF700	CD8	HIT8a	Biolegend	300919
	15	APC-Cy7	Zombie NIR	---------	Biolegend	423105
**B cells**	1	BV570	IgM	MHM-88	Biolegend	314516
	2	BV785	CD27	O323	Biolegend	302831
	3	PE	CD19	SJ25C1	Biolegend	363003
	4	PerCP-Cy5.5	CD38	HB-7	Biolegend	356613
	5	PE-Cy7	CD21	Bu32	Biolegend	354911
	6	APC	IgG	M1310G05	Biolegend	410711
	7	AF700	CD138	MI15	Biolegend	356511
	8	APC-Cy7	Zombie NIR	---------	Biolegend	423104

**AIM assay**	1	BV421	CD45RA	HI100	Biolegend	304129
	2	BV510	PD-1 (CD279)	EH12.2H7	Biolegend	329931
	3	BV570	CD4	RPA-T4	Biolegend	317445
	4	BV605	CXCR5	J252D4	Biolegend	356930
	5	BV711	CD25	M-A251	Biolegend	356137
	6	FITC	CD69	FN50	Biolegend	310904
	7	PE	PD-L1 (CD274)	29E.2A3	Biolegend	329705
	8	PE-Dazzle 594	CD3	UCHT1	Biolegend	300450
	9	PE-Cy5	CD40L	24-31	Biolegend	310808
	10	PerCP-Cy5.5	OX40/CD134	Ber-ACT35	Biolegend	350009
	11	PE-Cy7	CXCR3	G025H7	Biolegend	353719
	12	AF700	CD8	HIT8a	Biolegend	300919
	13	APC-Cy7	Zombie NIR	---------	Biolegend	423105
**Cytokines**	1	BV421	TGF-β1 (LAP)	TW4-2F8	Biolegend	349613
	2	BV510	TNF-α	MAb11	Biolegend	502949
	3	BV570	HLA-DR	L243	Biolegend	307637
	4	BV785	CD14	63D3	Biolegend	367141
	5	FITC	IL-1β	JK1B-1	Biolegend	508206
	6	PE	IL-23p19	727753	Invitrogen	MA5-23644
	7	PE-Dazzle 594	IL-6	MQ2-13A5	Biolegend	501121
	8	PerCP-Cy5.5	IL-12p40	C11.5	Biolegend	501821
	9	PE-Cy7	IL-27p28	MM27-7B1	Biolegend	516910
	10	APC	TLR8 (CD288)	S16018A	Biolegend	395506
	11	AF700	IL-18	74801	R&D Systems	IC646N-100UG
	12	APC-Cy7	Zombie NIR	---------	Biolegend	423105

Supplementary Legends

Supplementary Figure 1. *In vitro* TLR8 stimulation augments proinflammatory cytokines in monocytes. Representative flow plots show (**A**) sequential gating strategy for detecting cytokine positive monocytes. The bar graphs indicate (**B**) frequencies of $CD14^{+}HLA\text{-}DR^{+}$ monocytes in response to various treatments, (**C**) TLR8 expression in response to various treatments and frequencies of $CD14^{+}HLA\text{-}DR^{+}$ monocytes producing (**D**) TGF-β1 and (**E**) IL-27p28. Error bar on the graph represents the standard error of mean calculated from 6 individual CHB patient PBMCs. Differences between stimulations were calculated by one-way analysis of variance (ANOVA) or Kruskal-Wallis test for multiple comparisons. P values of ≤0.05* was considered statistically significant.

Supplementary Figure 2. TLR8-specific agonism upregulated HBV-specific GC-like T_{FH} development factors and their subsets. Flow cytometry plots show (**A**) gating strategy for T_{FH} panel and (**B**) CXCR3/CCR6 cT_{FH} subsets compared between untreated (UT/control), TLR8 stimulations ssRNA40, TL8-506, PMA/Ion, ssRNA40 stim and TL8-506 stim. “Stim” means subtraction of PMA/Ion from the respective values of TLR8 + PMA/Ion re-stimulations. Frequencies of circulating follicular helper T (cT_{FH}) cells and their subsets are shown in (**C**) $CXCR5^{+}CD4^{+}$ cT_{FH} cells gated on $CD3^{+}$ T lymphocytes, (**D**) $cT_{FH}1$ ($CXCR3^{+}CCR6^{-}$), (**E**) $cT_{FH}2$ ($CXCR3^{-}CCR6^{-}$), (**F**) $cT_{FH}17$ ($CXCR3^{-}CCR6^{+}$) and (**G**) $cT_{FH}1/17$ ($CXCR3^{+}CCR6^{+}$) gated on $CXCR5^{+}CD4^{+}$ cT_{FH} cells. Each symbol in the graph shows 6 individual donors and horizontal bar in the graph represents median range values. Numbers presented inside the box indicate percentages. Square quadrants diagram illustrate circulating T_{FH} subsets. The differences

between stimulations were evaluated by one-way ANOVA or Kruskal-Wallis test for multiple comparisons. P values of ≤0.05*, 0.01**, 0.001*** indicate significance.

Supplementary Figure 3. Selgantolimod treatment enhanced T_{FH} differentiation factors in CHB clinical samples. Selective-TLR8 agonist Selgantolimod was orally administered to patients infected with CHB and PBMCs (n=8-10) were tested from baseline (BL, pre) and TLR8 (8h post-Selgantolimod). PBMCs were left untreated (UT) or treated with combination of HBsAg/HBV Pep for 5 days followed by re-stimulation with PMA/Ion. (**A-F, H**) represents flow plots and frequencies of co-expression of IL-21^{+}BCL-6^{+}, ICOS^{+}BCL-6^{+}, ICOS^{+}CD40L^{+} and PD-1^{+}CXCR3^{-} cells, gated on CXCR5^{+}CD4^{+} cT_{FH} cells (**G**) upregulated with TLR8 agonist (8h post-single oral dose). Numbers inside boxes indicate percentage of double positive cells. A two-tailed paired, non-parametric or the Wilcoxon signed-rank Student's t test were conducted to evaluate the differences between BL and 8h post-TLR8 treated samples. P values of ≤0.05* indicates statistical significance. CHB, chronic hepatitis B; HBV Pep, HBsAg/HBV peptides pool; PMA, phorbol-12-myristat-13-acetate; Ion, ionomycin; ns, no significance.

Supplementary Figure 4. Selgantolimod rescued the defective HBV-specific activation induced markers (AIM) of GC-like T_{FH} cells in Phase 1B clinical subjects. FACS analysis was performed using AIM assay panel antibodies (Supplementary Table 1). Flow plot represents (**A**) gating strategy for AIM assay panel. Frequencies of (**B**) cT_{FH} cells (CXCR5^{+}CD4^{+} gated on CD3^{+} T lymphocytes) and (**C**) GC-like T_{FH} (PD-1^{+}CXCR3^{-} cells gated on CXCR5^{+}CD4^{+} cT_{FH} cells) cells are compared between HBV vaccinated HC, BL and TLR8 (8h post-Selgantolimod) after *in vitro* stimulations with HBV pep or SEB. Each symbol in the graph shows individual

donor and horizontal bar in the graph represents median range values. A two-tailed unpaired or paired, non-parametric or the Wilcoxon signed-rank Student's t tests were conducted. P values of ≤0.05*, 0.01**, 0.001*** indicate significance.

Supplementary Figure 5. TLR8 signaling increased memory B, plasma B and modulated B cell subsets. Flow plots indicating (**A**) sequential gating strategy of B cell panel and (**B**) $CD19^+$ B lymphocytes. Frequencies of (**C**) B cells ($CD19^+$) (**D**) PCs ($CD19^-CD138^+$) and (**E**) PBs ($CD38^+CD138^-$ cells gated on $CD19^+CD27^{++}$ B lymphocytes) are shown. Dotted line distinguishes the data between TLR8 without or with HBV Pep treatments. Statistical significances were performed by two-tailed, non-parametric or the Wilcoxon signed-rank Student's t test for comparison between stimulations. P values of ≤0.05*, 0.01** were statistically significance. ns, no significance; PCs, plasma cells; PBs, plasmablasts.

Supplementary Figure 6. TLR8 signaling promotes IL-12 dependent GC-like T_{FH} cell differentiation and improved IL-21 production. Autologous naïve $CD4^+$ T cells were isolated from PBMCs of HC (n=2) and CHB patients (n=2) and co-cultured with $CD14^+$ enriched monocytes (as APCs, 2:1 ratio) previously stimulated with medium (UT, negative control), TLR8-specific ligands ssRNA40 or TL8-506 along with isotype control IgG, anti-IL-12 or anti-IL-18 neutralizing (blocking) Ab and cultured for 6 days in the presence of SEB followed by re-stimulation with PMA/Ion to activate T_{FH} cells. Flow cytometry evaluation with T_{FH} panel markers using manufacturer protocol as stated in Figure 2 shows (**A**) intracellular expression of IL-21, gated on (**E**) cT_{FH} ($CD3^+CD4^+CXCR5^+$ T) cells. Co-expression of GC-like T_{FH} differentiation markers (**B**) $IL\text{-}21^+ICOS^+$ and (**C**) $ICOS^+$PD-1+ on $CD3^+CD4^+CXCR5^+$ T cells

(**D**). (**F**) Intracellular expression of IL-21 producing CXCR5+ cells, generated from naïve $CD4^+$ T cells (without monocytes as APCs) cultured with medium (UT), rhIL-12 or rhIL-18 for 6 days followed by re-stimulation with PMA/Ion. Numbers inside the quadrants represents percentage of events. rhIL-12 and rhIL-18, recombinant human protein IL-12 and IL-18; Cont. IgG, isotype control IgG.

Supplementary Figure 7. Flow plots (**A**) represent the co-expression of IL-21^+CXCR5^+, IL-21^+BCL-6^+ and $ICOS^+$BCL-6^+ markers, gated on CXCR5^+CD4^+ cT_{FH} cells after stimulation of CpG-ODN (non-TLR8 agonist) and PMA/Ion for 5 days. Representative flow plots also show comparative co-expression of (**B**) OX40^+CD25^+, (**C**) PD-L1^+CD25^+ and (**D**) CD69^+CD40L^+ cells gated on CXCR5^-CD4^+ T cells in HC and CHB (BL and TLR8) samples. Numbers shown inside the flow box indicate percentage of events.

Supplementary Figure 8. PBMCs from CHB patients (n=3) were left untreated (UT) or treated with TLR8 agonists ssRNA40 or TL8-506 for 5 days. ssRNA40 stim and TL8-506 stim indicates subtraction of overnight PMA/Ion stimulations from the respective values of TLR8 + PMA/Ion re-stimulations. Illustrative flow plots show (**A**) intracellular expression of IFNγ and TNFα, gated on cT_{FH} (CXCR5^+CD4^+CD3^+ T lymphocytes) cells. (**B**) Represents FMO controls on the expression of IL-21, ICOS and BCL-6 cells, gated on CXCR5^+CD4^+ cT_{FH} cells after 5 days culture of PBMCs from CHB patients (n=3) with ssRNA40 stim.

Supplementary Figure 1

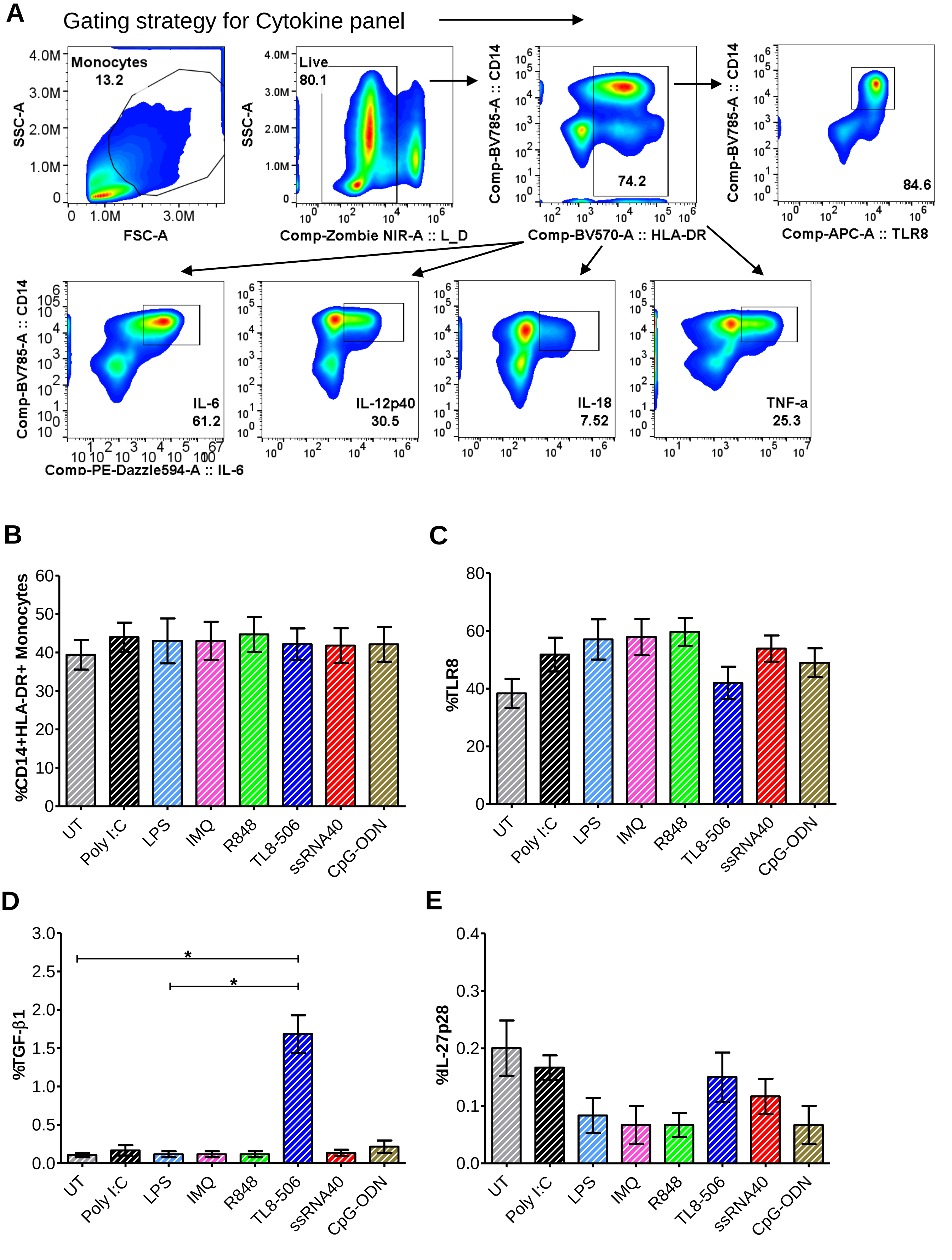

Supplementary Figure 2

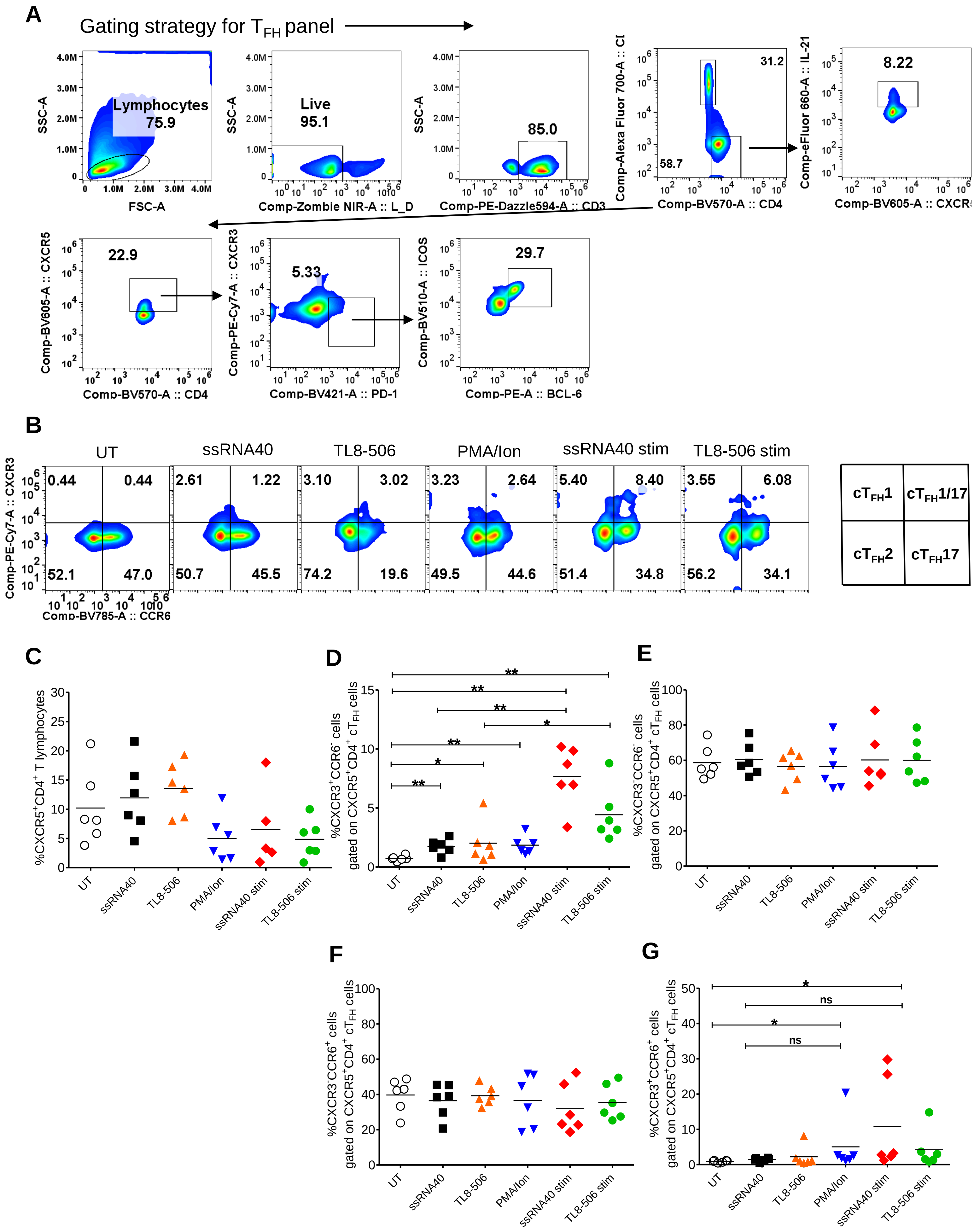

Supplementary Figure 3

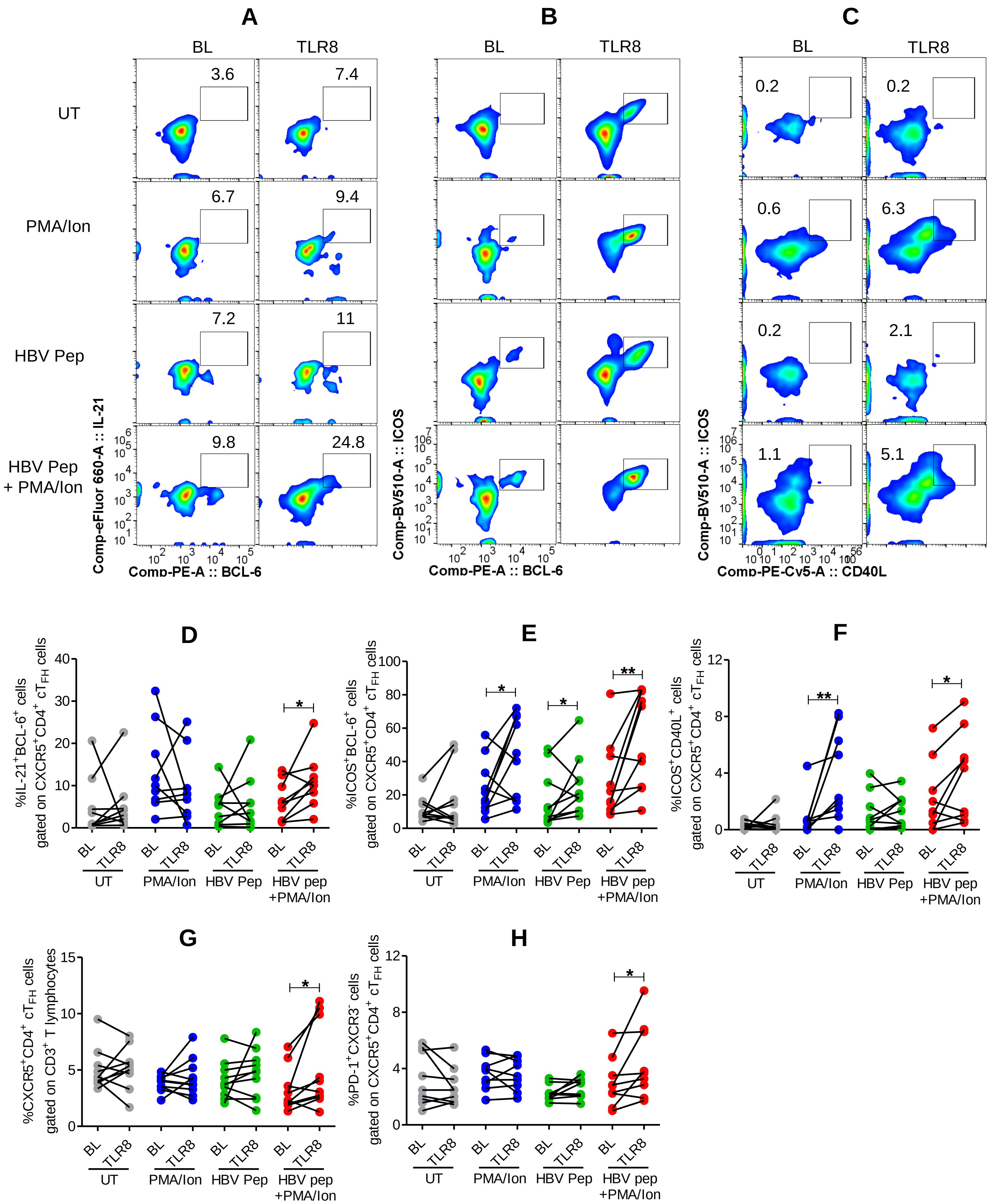

Supplementary Figure 4

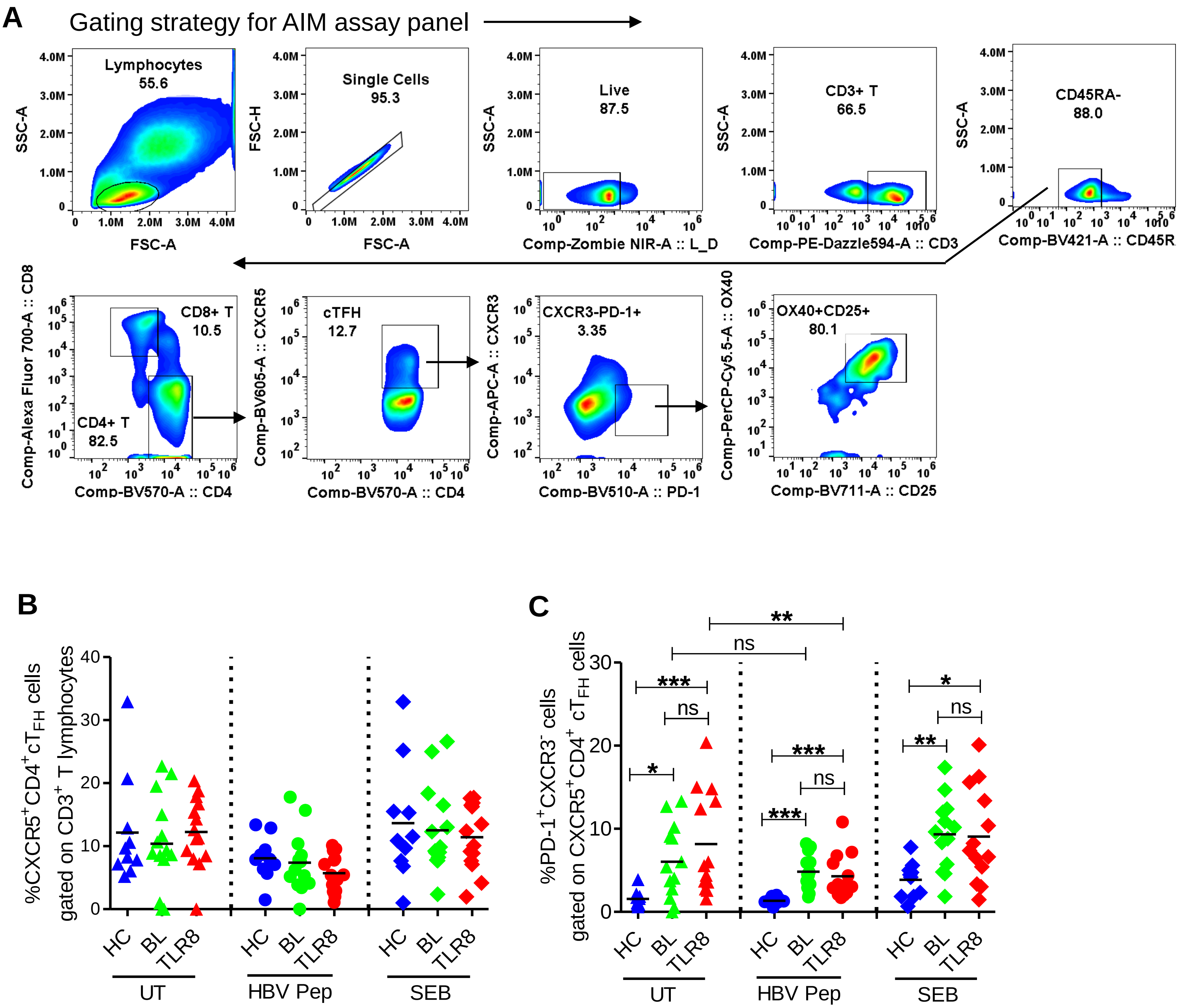

Supplementary Figure 5

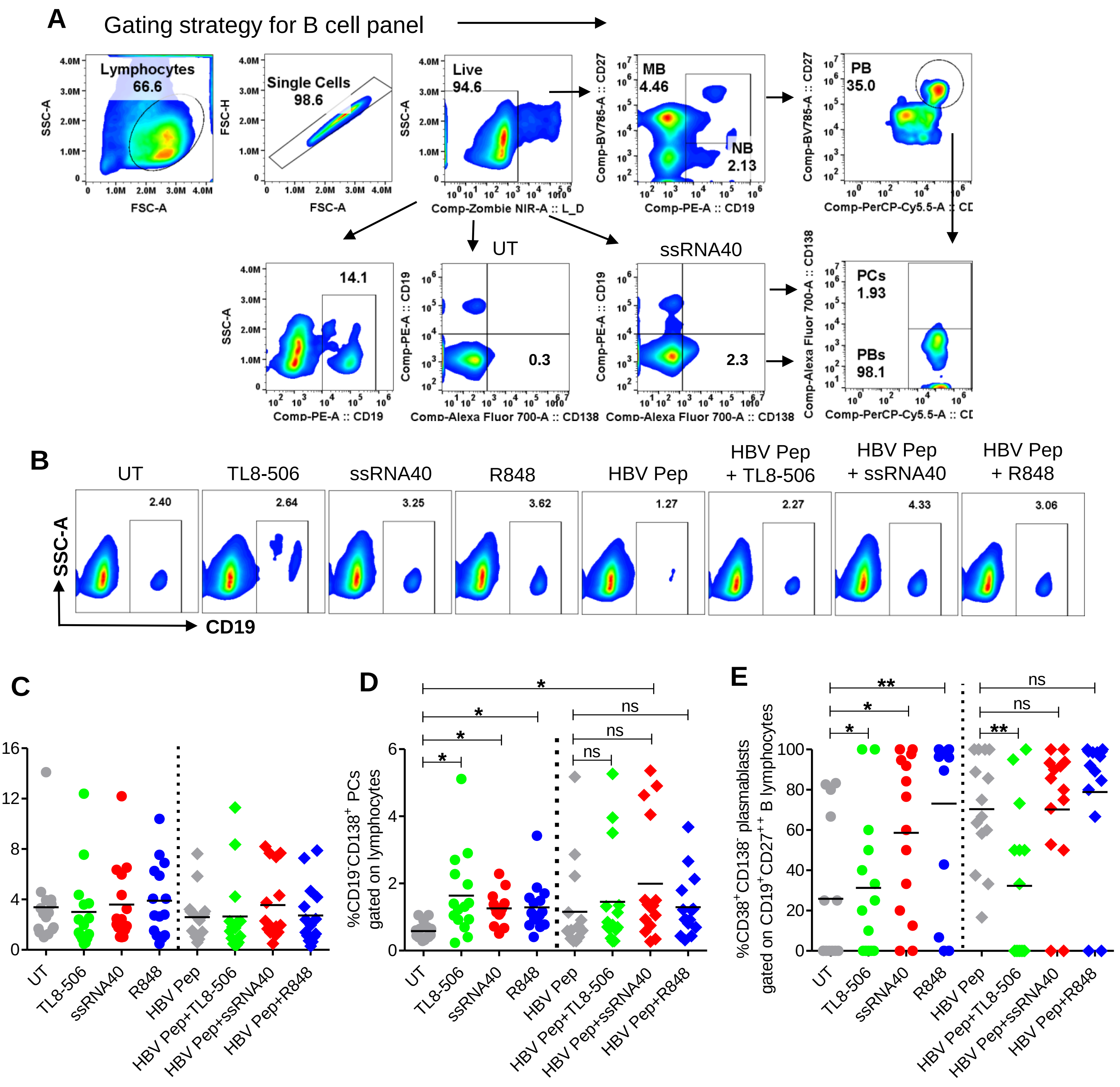

Supplementary Figure 6

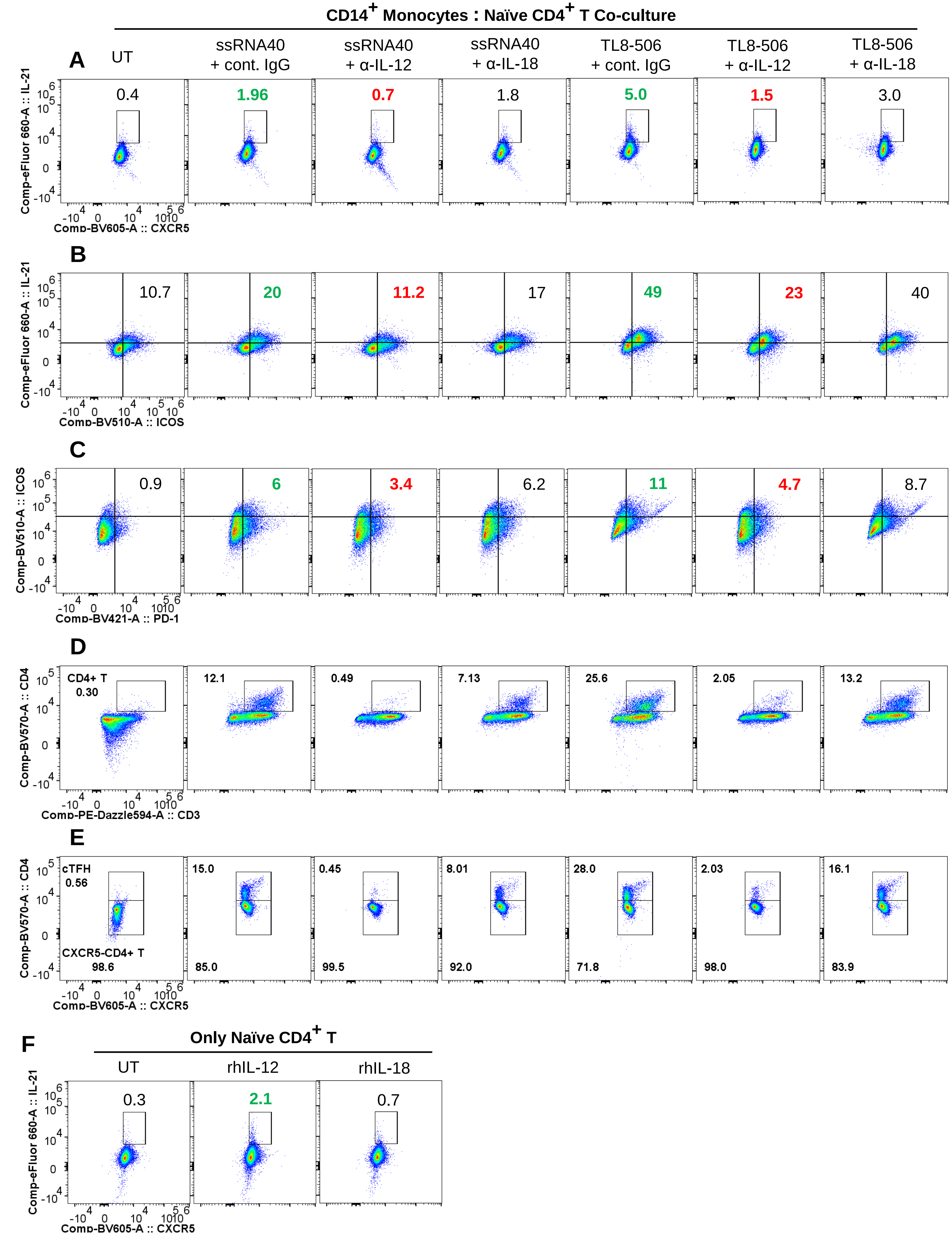

Supplementary Figure 7

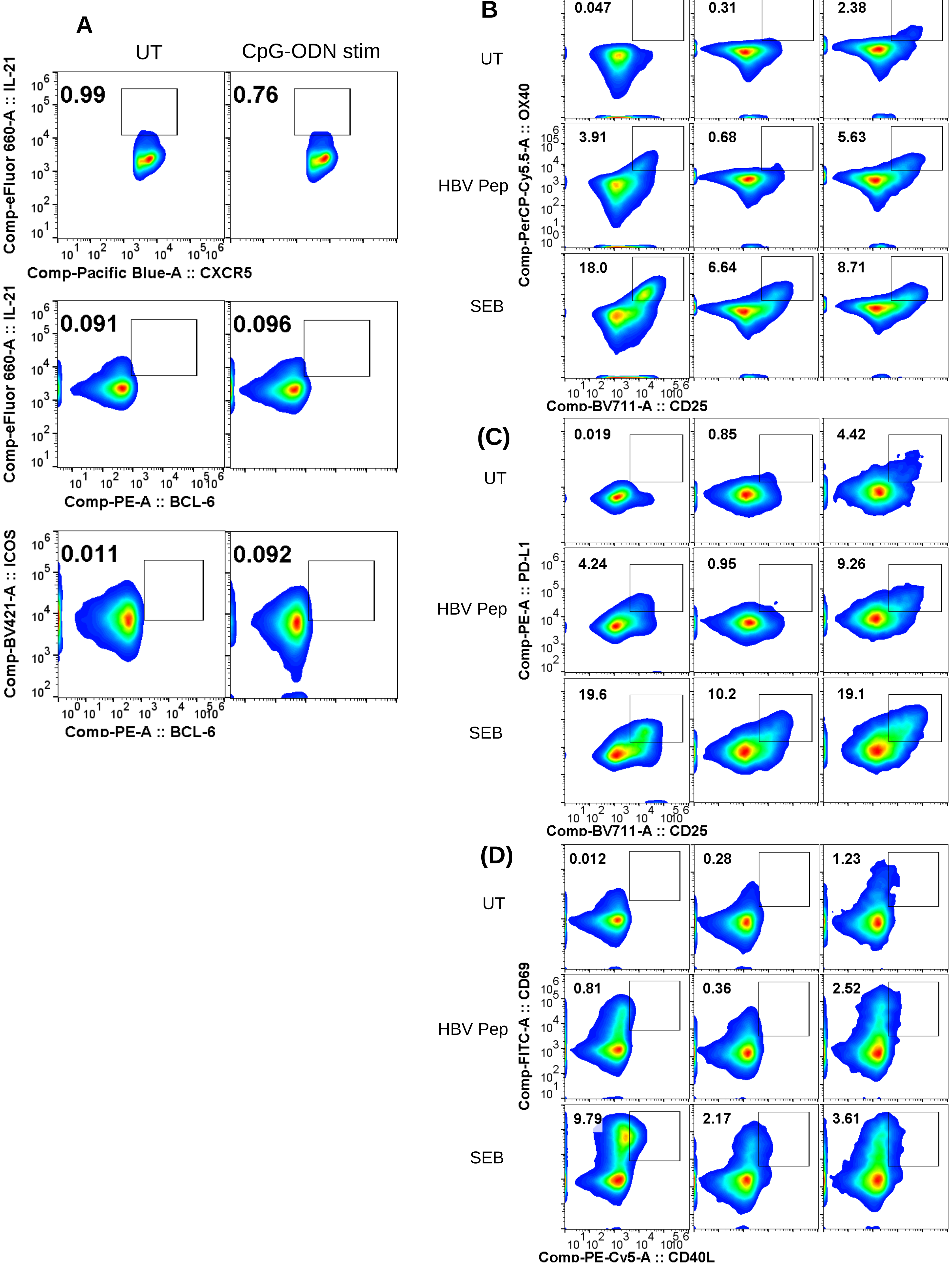

Supplementary Figure 8

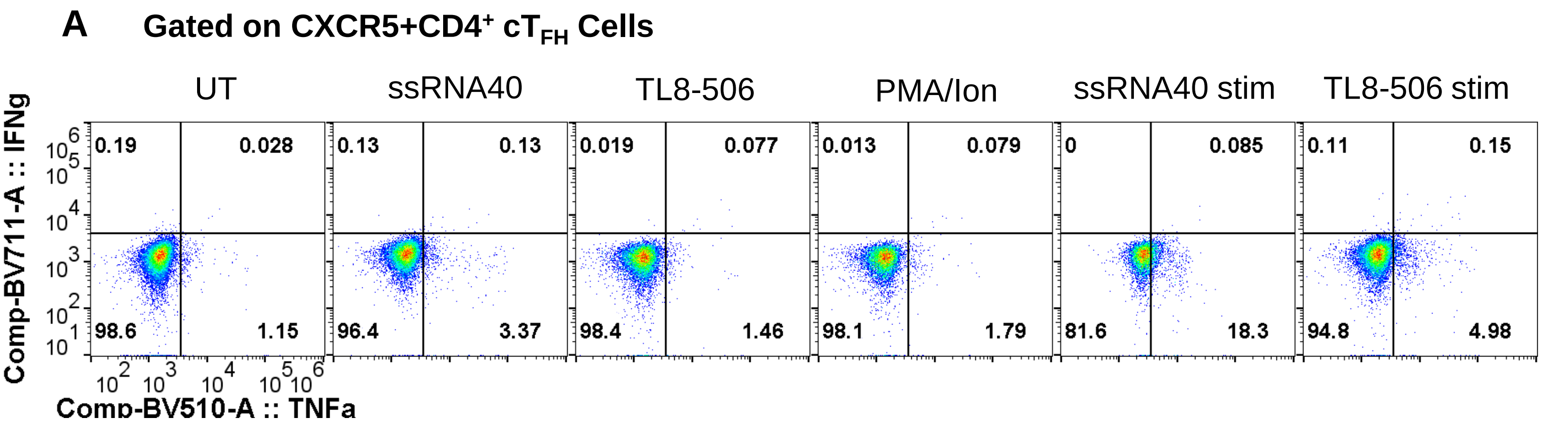

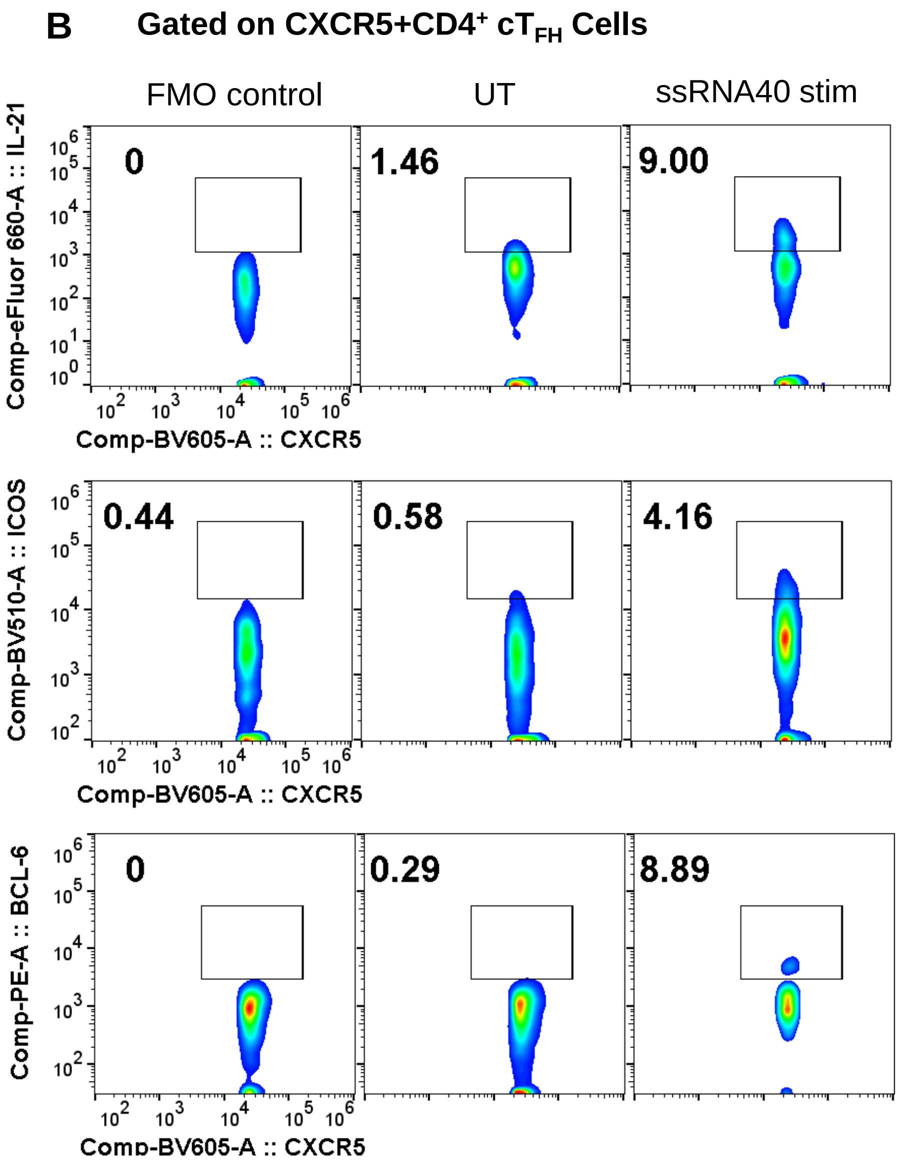

Supplement: Supplementary file 1 [file DataSheet_1.pdf]
